# A Review on the Different Aspects and Challenges of the Dry Reforming of Methane (DRM) Reaction

**DOI:** 10.3390/nano12193400

**Published:** 2022-09-28

**Authors:** Aseel G. S. Hussien, Kyriaki Polychronopoulou

**Affiliations:** 1 Department of Mechanical Engineering, Khalifa University of Science and Technology, Main Campus, Abu Dhabi P.O. Box 127788, United Arab Emirates; 2 Center for Catalysis and Separations (CeCaS), Khalifa University of Science and Technology, Abu Dhabi P.O. Box 127788, United Arab Emirates

**Keywords:** dry reforming of methane, catalysts, ceria, DFT

## Abstract

The dry reforming of methane (DRM) reaction is among the most popular catalytic reactions for the production of syngas (H_2_/CO) with a H_2_:CO ratio favorable for the Fischer–Tropsch reaction; this makes the DRM reaction important from an industrial perspective, as unlimited possibilities for production of valuable products are presented by the FT process. At the same time, simultaneously tackling two major contributors to the greenhouse effect (CH_4_ and CO_2_) is an additional contribution of the DRM reaction. The main players in the DRM arena—Ni-supported catalysts—suffer from both coking and sintering, while the activation of the two reactants (CO_2_ and CH_4_) through different approaches merits further exploration, opening new pathways for innovation. In this review, different families of materials are explored and discussed, ranging from metal-supported catalysts, to layered materials, to organic frameworks. DRM catalyst design criteria—such as support basicity and surface area, bimetallic active sites and promoters, and metal–support interaction—are all discussed. To evaluate the reactivity of the surface and understand the energetics of the process, density-functional theory calculations are used as a unique tool.

## 1. Introduction

**Figure 1:**
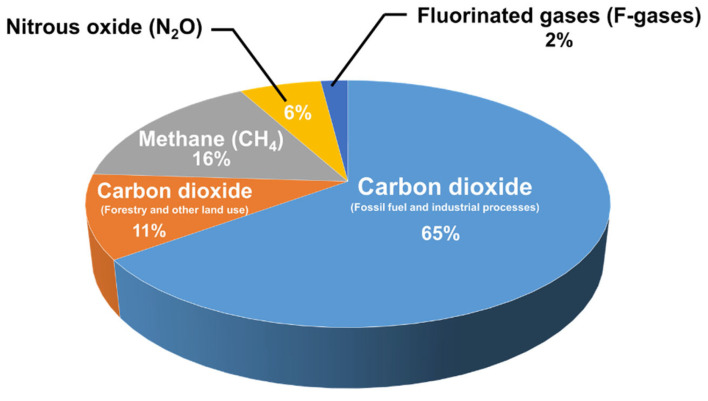
Global greenhouse gas (GHG) emissions based on global emissions from 2010 reported in the Contribution of Working Group III to the Fifth Assessment Report of the Intergovernmental Panel on Climate Change [4].

The continuous increase in global energy demand has resulted in the depletion of fossil fuels and an increase in carbon dioxide (CO_2_) emissions. In light of the increasing catastrophic effects of greenhouse gases (GHGs) on the Earth’s environment, many countries (195) signed an agreement in 2015 under the United Nations Framework Convention on Climate Change (UNFCCC) to reduce the detrimental effects of global warming [1,2]. The ultimate goal of this agreement is to decrease the global temperature increase, subsequently leading to major changes in the energy sector and adopting greener and more sustainable energy sources [1]. Studies have shown that the largest GHG contributor is carbon dioxide, followed by methane (CH_4_), as shown in the reported global emissions pie chart in Figure 1 [3,4]. This has also led to the flourishment of the decarbonization industry, which deals with the capture and utilization of CO_2_, as well as activities such as the synthesis of more valuable chemicals [2,5]. At the same time, the abundance of natural gas reserves [6] and the CH_4_-rich composition of biogas (i.e., 45–55%)—the latter produced from the anaerobic digestion of organic matter—make the dry reforming of methane (DRM) reaction a vehicle for the simultaneous transformation of both CH_4_ and CO_2_ (Equation (2)). The DRM converts two greenhouse gases to synthesis gas (syngas, CO and H_2_) [3] at a ratio that favors the long-chain hydrocarbons and value-added products; this was the motivation of this review article.

Despite the tremendous value of the DRM reaction, as stated above, this process has not yet been industrialized, due to the absence of an economically feasible catalyst that does not suffer from coking and sintering. This review article highlights all of the aspects of DRM, with emphasis on the catalyst design criteria and the understanding of the intrinsic catalyst characteristics in pursuit of a sustainable catalyst system. The benefits of DRM are further discussed in Section 2, followed by a brief on the kinetics and thermodynamics of the DRM reaction. The challenges faced in DRM industrialization are discussed in Section 3. A plethora of studies conducted on tuning the properties for a wide range of materials to enhance their catalytic performance for the DRM are presented in Section 4, where isotopic techniques are critically discussed. Additionally, computational studies on the DRM are discussed in Section 5. Concluding remarks and future perspectives are provided in Section 6.

## 2. Natural Gas and Reforming Technologies for Syngas Production

**Figure 2:**
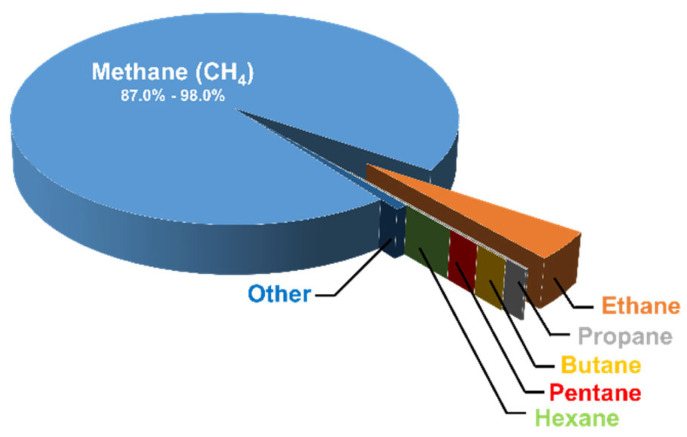
Composition of natural gas obtained from [7], which may vary depending on the extraction location (based on data from the website).

Natural gas is a mixture of hydrocarbon gases that consists mainly of methane, as illustrated in the pie chart below (Figure 2) [7]. The type of organic material and the geological formation of the reservoir rock from which the natural gas was obtained affect the percentages of the constituents of natural gas. In recent years, natural gas has attracted the attention of researchers, and this can be attributed to the fact that there is a vital need to find greener fuels capable of replacing petroleum-based fuels. In addition, natural gas is an ideal input for more valuable products. Although non-renewable petroleum reserves are overexploited, natural gas reserves are not sufficiently utilized, because they are not considered as valuable as petroleum reserves. Sometimes, natural gas is even burned because it is considered as an undesired byproduct. However, this is changing due to the increasing demand for hydrogen production from hydrocarbons, and it was found by Fishtil et al. that natural gas is most suitable [8]. This is due to the fact that the major constituent of natural gas (methane) is considered to be the most appropriate hydrocarbon to obtain hydrogen through reforming or partial oxidation reactions, since it has the highest ratio of hydrogen to carbon (H:C); the latter is useful to decrease the emissions of the greenhouse gas carbon dioxide (CO_2_). Furthermore, natural gas is abundantly available [9]. Other fossil fuels can be used to produce hydrogen, such as coal via an integrated gasification combined cycle (IGCC). Coal has the largest reserves of all fossil fuels worldwide, but it has a low H:C ratio. Therefore, it produces more CO_2_, making it necessary to use energy-demanding separation and capturing processes. Consequently, hydrogen production from coal is more expensive, and is avoided by industries [9].

**Figure 3:**
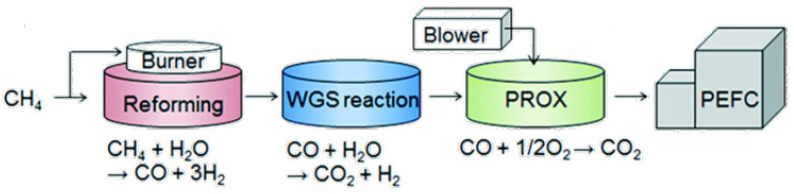
Catalytic processes used sequentially to obtain high-purity hydrogen for PEFC systems, where WGS represents water–gas shift reaction, PROX indicates preferential reaction oxidation of CO, and PEFC represents polymer electrolyte fuel cells. Reprinted with permission from Ref. [12]. Copyright 2015 Royal Society of Chemistry.

Hydrogen fuel can be produced from methane using natural gas reforming, also known as reforming of methane. This is a catalytic process used to convert methane into syngas (H_2_ + CO). Syngas can be used to produce hydrogen and other important petrochemicals, such as ammonia (NH_3_) and methanol (CH_3_OH). Then, the syngas undergoes further chemical processing to remove CO and obtain hydrogen [10,11]. The following sequential catalytic processes are used to obtain high-purity hydrogen: (i) natural gas reforming, (ii) water–gas shift reaction (WGSR), and (iii) preferential oxidation of CO (PROX), as illustrated in Figure 3.

Natural gas reforming can be performed through four main chemical reactions: (i) steam reforming, (ii) dry reforming, (iii) partial oxidation, and (iv) autothermal reforming. These processes differ in the process parameters used, such as the oxidant (H_2_O, O_2_, or CO_2_), H_2_:CO ratio, kinetics, and energetics of the reaction. In the followi8ng text, the main characteristics of each process are discussed in brief.

### 2.1. Steam Reforming

This is an endothermic reaction (Equation (1)), where steam is the oxidant, and requires a lot of energy (operating temperature ~850 °C) to obtain desirable conversion [13], making this process expensive. CH_4_ reacts with H_2_O in the presence of a catalyst to produce syngas with a H_2_:CO ratio of 3:1 [10,11,14]. The high cost of this reaction makes researchers focus on exploring other processes.

(1) $$CH_{4}+H_{2}O\to CO+3H_{2},\;\Delta H_{o}=206\;kJmol^{-1}$$

### 2.2. Dry Reforming of Methane (DRM)

DRM is an endothermic reaction that uses carbon dioxide to reform methane to syngas (Equation (2)). This endothermic reaction is conducted at high temperatures (>700 °C) [15]. The syngas produced has a H_2_:CO ratio of 1:1 [10,11,14], as illustrated in the equation below. DRM is a bifunctional reaction, implying that both the metal and the support should be tailored so as to suppress any parasitic reactions and favor the reaction itself. The increase in coke formation is attributed to the use of CO_2_ as a reagent [9].

(2) $$CH_{4}+CO_{2}\to 2CO+2H_{2},\;\Delta H_{o}=247.3\;kJmol^{-1}$$

#### 2.2.1. Brief on DRM Thermodynamics

The DRM reaction is a slow reaction that requires a large amount of energy (heat) to dissociate the highly stable molecules CO_2_ (526 kJ mol^−1^) and CH_4_ (435 kJ mol^−1^) to reach equilibrium and convert them to synthesis gas, resulting in long conversion times and, thus, introducing challenges in its industrialization [16]. Additionally, side reactions occur simultaneously with DRM that affect the equilibrium of the reaction, such as reverse water–gas shift (RWGS: CO_2_ + H_2_ → CO + H_2_O, ΔH_o_ = +41 kJ mol^−1^). This increases the conversion of CO_2_, resulting in a syngas with a H_2_/CO ratio below 1. Other than RWGS, carbon is formed through two other main pathways, namely, (i) CH_4_ decomposition reaction, and (ii) Boudouard or CO disproportionation reaction, which causes the deactivation of the catalyst by blocking the metal active sites. The role of these two reactions in coking is thoroughly discussed in the following sections.

**Figure 4:**
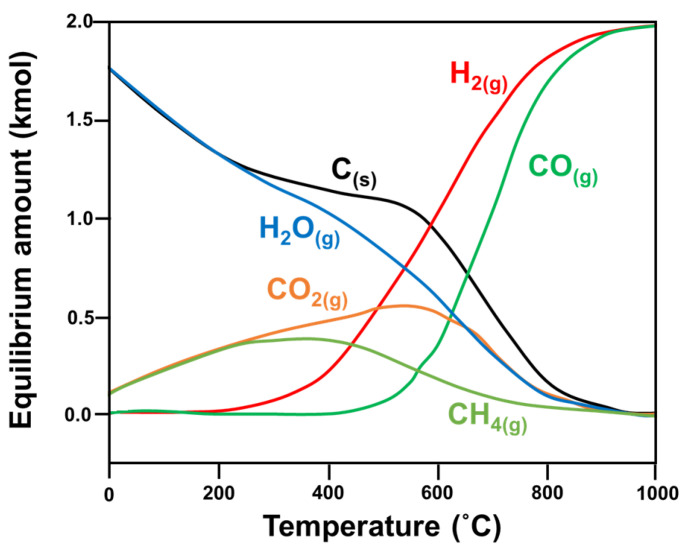
Equilibrium data for the DRM process. Reprinted with permission from Ref. [6]. Copyright 2016 Royal Society of Chemistry.

Research has shown that CH_4_ decomposition occurs at temperatures higher than 557 °C, while the CO disproportionation reaction occurs at temperatures below 700 °C. Hence, carbon formation occurs in the 557–700 °C temperature range, and the optimal operational temperature for DRM is 870–1040 °C, with a feed ratio of CO_2_/CH_4_ equivalent to 1 [17]. Other studies have used varied reaction parameters—such as temperature [17,18,19,20,21], pressure [17,19], CO_2_/CH_4_ feed ratio, and addition of another oxidant—to find the optimal conditions to minimize the Gibbs free energy (ΔG) of the reaction (Figure 4 [6]).

The thermodynamic relationship is defined (Equation (3)) in terms of equilibrium constant (K_p_), partial pressure (P_i_), Gibbs free energy (ΔG°), temperature (T), pressure (P), molar fraction (y), stoichiometric ratio (ν), and gas constant (R).

(3) $$\prod \left(y_{i}P\right)\nu_{i}=e^{\frac{\Delta G^{{}^{\circ}}}{RT}}=F\left(\alpha \right)$$

where

$$y_{i}=\frac{n_{i0}+\nu_{i}\propto }{n_{0}+\nu \propto }$$

$n_{i0}$ = number of moles of the *i*th species;

$n_{0}$ = number of moles of the reaction.

The extent of change in the reaction (∝) with respect to the operating parameters can be analyzed using Equation (4).

(4) $$\frac{d\alpha }{dy}=\frac{CF\left(\alpha \right)}{F\left(\alpha \right)}$$

where C is equivalent to $\left(\frac{\Delta H}{RT^{2}}\right)$ if the extent of change in the reaction (∝) is analyzed with respect to temperature, and C is equivalent to $\left(\frac{-\vartheta }{P}\right)$ if the extent of change in the reaction (∝) is analyzed with respect to pressure.

Since DRM is an endothermic reaction, *C* is negative when the extent of the reaction (∝) is analyzed with respect to temperature (T), and the reaction is favored at high temperatures. Meanwhile, in the case of exothermic reactions, *C* is positive, and low temperatures are favored. Reactions with decreasing moles with positive *C* favor higher pressures, and vice versa [6].

#### 2.2.2. Brief on DRM Kinetics

The kinetics of DRM are similar to those of steam reforming of methane (SRM), as reported by the findings of Bodrov and Apelbaum. However, in Bodrov’s model, the oxidant assumed was steam and not CO_2_ [22]. Later, Zhang and Verykios derived a Langmuir–Hinshelwood (LH) model for DRM over a Ni-based catalyst supported on CaO-Al_2_O_3_, and considered that CH_4_ was the rate-determining step (RDS) [23]. On the other hand, Slagtern et al. [24] suggested that both CH_4_ and CO_2_ dissociation are the RDSs. The CH_4_ dissociation forms carbon species, while CO_2_ dissociation provides oxygen. Hu and Ruckenstein [25], as well as Slagtern et al. [24], concluded that the reactions that occur on the surface of the Ni catalyst between oxygen and carbon educts are RDSs. Most of the kinetic models proposed for DRM propose an LH model [26]. Wei and Iglesia [27] performed an isotopic kinetic study, and concluded that the activation of the C-H bond is the only kinetically relevant step for DRM over Ni-based catalysts. Other researchers argue that the surface reaction between CH_x_* and O* species is the RDS [28] over Ni-based catalysts during DRM. Other researchers indicate the carbon oxidation as the RDS [29,30,31], while still others conclude that CH_x_O decomposition is the RDS on Ni catalysts during DRM [32,33]. Wang et al. surmised that these varying conclusions could be attributed to the fact that the reaction mechanism is a function of the operating conditions. A mechanistic study over a Ni/SiO_2_ catalyst was performed by Kroll et al. [34], using non-steady-state and steady-state isotopic transient experiments combined with in situ DRIFTS. It was found that immediate contact with the reactants caused instantaneous formation of carbon species on the surface of the catalyst, originating from CH_4_ and CO_2_ dissociation on the Ni active sites. Isotopically labelled deuterated methane (CD_4_) was used to prove the reversibility of the methane activation step (CH_4_ + S_1_ ↔ C_ads_ + 2H_2_) at 700 °C. However, the oxidation step (C_ads_ + O_ads_ → CO + S_1_ + S_2_) which was studied via SSITKA, temperature-programmed oxidation (TPO), and temperature-programmed hydrogenation (TPH), was found to be the RDS. On the other hand, CH_4_ activation exhibited no kinetic isotopic effect; hence, it is not the RDS. The same research group used SSITKA and temporal analysis of products (TAP) experiments to mechanistically study DRM on Ni and Ru supported on SiO_2_ [35]. The same conclusion was deduced—that the RDS does not involve the C–H bond activation (fast step), and the RDS is the slow oxidation step. Additionally, Chang et al. [36] used deuterium isotopic experiments (CD_4_) to mechanistically study DRM over a KNiCa catalyst. The study confirmed the findings of the previous [34,35] mechanistic studies over Ni-based catalysts—that CH_4_ dissociation is not the RDS, while the reaction between the carbon adsorbed and the dissociated oxygen on the Ni active sites to produce CO is the RDS.

As mentioned above, some studies report that the surface reaction between CHx* and O* is the RDS [28] over Ni-based catalysts [32,33]. In particular, a study was performed by Lou et al. [32] on a Ni-based catalyst (Ni-La_2_O_3_/5A molecular sieve) synthesized via the citric acid complexing method. Numerous experiments were utilized to investigate the RDS and the mechanisms of DRM over the synthesized catalyst, such as CD_3_I chemical trapping experiments, ^13^CH_4/_CO_2_ pulse experiments, etc. The tests showed that the decomposition of CHO and CH_2_O is the RDS. In addition, Osaki et al. examined the kinetics of DRM over K-Ni/Al_2_O_3_ catalysts with different K wt.% (0, 1, 5, and 10 wt.%). The presence of K improved carbon inhibition by controlling the size of the Ni particles. Their results showed that the dissociation of CH_x_O_ads_ is the RDS (CH_x_O_ads_ → CO + x/2H_2_). The rate of this reaction exhibited no change when the surface coverage of K was below the threshold value (Θ_K_ = 0.4). On the other hand, when Θ_K_ was greater than 0.4, the rate of the reaction slowed down; this was attributed to the blockage of Ni active sites by K particles.

Another reaction model proposed by Bradford and Vannice [37] suggested CH_4_ dissociation and CH_x_O decomposition as RDSs. They were even able to correlate their results with their experimental findings. The same conclusions were deduced by Nandini et al. [38] over a Ni–K/CeO_2_–Al_2_O_3_ catalyst. The kinetic study showed that temperature affects the RDS [39]. In the case of low temperatures, CH_4_ dissociation was the RDS, while at high temperatures the RDS was the reaction between CO_2_ and CH_x_.

DRM is a reversible reaction, and determining the equilibrium constant K using the kinetic rate constants of forward and backward reactions can indicate which reaction is more favorable [6]. This also applies to the side reactions that occur simultaneously with DRM. Generally, in the case of reversible reactions, the reaction rates of the forward (r_f_) and reverse (r_r_) reactions, along with their respective activation energies (E_a_), can be determined from the Arrhenius relation of the thermodynamics to the kinetics, as shown in Equation (5):

(5) $$k=k_{0}exp^{\frac{-E_{a}}{RT}}$$

where, $k_{0}$ is the pre-exponential factor, T is the temperature, and R is the universal gas constant.

The equilibrium constant K is derived from the ratio of the forward reaction rate constant (*k*_f_) to the reverse reaction rate constant (*k_r_*), which are presented below in Equations (6) and (7), respectively. The equilibrium constant determines the extent of the DRM reaction [21]. In reversible reactions, the molar ratio has a great effect on the distribution of products when K is close to unity. However, molar ratios of reactants have no effect if K >> 1 [16]. Additionally, DRM can occur spontaneously if the activation energy (ΔE_a_) is negative.

(6) $$k_{f}=k_{0f}exp^{\frac{-E_{a}}{RT}}$$

(7) $$k_{r}=k_{0r}exp^{\frac{-E_{a}}{RT}}$$

### 2.3. Partial Oxidation

Partial oxidation (POX) is considered more economical than the two processes (SRM and DRM) mentioned above, because it is an exothermic reaction. The industrial operating conditions of partial oxidation of methane to syngas are at temperatures higher than 920 °C and pressures above 800 kPa [40]. This reaction produces syngas with a ratio of H_2_:CO of 2:1 [14,41], as shown below (Equation (8)). However, it imposes a serious safety hazard, as there is a high risk of explosion [42].

(8) $$CH_{4}+\frac{1}{2}O_{2}\to CO+2H_{2},\;\Delta H_{o}=-36\;kJmol^{-1}$$

### 2.4. Autothermal Reforming

This process is a combination of two processes: steam reforming, and partial oxidation. Therefore, the reaction consists of three reagents: (i) methane, (ii) steam, and (iii) oxygen. In this reaction, the energy produced by the partial oxidation reaction is utilized to drive the endothermic steam reforming reaction [43,44]. The ratio of H_2_:CO of the syngas produced varies depending on the gaseous reactant fractions, so H_2_:CO can be 1:1 or 2:1 [45]. Typically, the temperature of the syngas produced via ATR ranges between 900 and 1100 °C [46]. The selection of the most appropriate reforming process depends on the economical aspect and the application in which the syngas used [9]. Scientists are trying to design the most ideal catalysts for these reactions to decrease the thermal consumption of these processes. Hence, it is important to understand which factors activate and deactivate catalysts, as discussed in the following section.

### 2.5. Critical Comparison of the Different Reforming Technologies

Steam reforming (SRM) is the most commonly used process, as it produces syngas with a high H_2_:CO ratio (3:1) that can be used to synthesize value-added chemicals, such as methanol and ammonia [9,40]. However, this reforming process produces CO_2_ with syngas, which increases the purification issue. Additionally, the H_2_:CO ratio of the syngas produced via steam reforming of methane is considered to be too high to be used for oxo-alcohol synthesis. H_2_:CO tuning is achieved through mixed reforming (O_2_/H_2_O/CO_2_) [40], whereas the H_2_:CO ratio of syngas produced via SRM is an undesirable ratio for Fischer–Tropsch (FT) synthesis. Carbon formation (coking) is another problem that SRM faces; even though the process is industrially mature technology, coke drastically deactivates the catalyst, reducing the performance in SRM [6]. The same issue is also faced under DRM. Other reforming methods use other oxidants, such as oxygen, e.g., the case of partial oxidation of methane (POM), which is exothermic. Hence, this is more economical than other endothermic reforming reactions (e.g., DRM and SRM). On the other hand, this process requires special equipment because it reaches as high as 1200 K [47], and needs an O_2_ supply plant, which is costly. Autothermal reforming (ATR) is a combination of SRM and POM, and it has been found to mitigate coking. The reactor used in ATR is considered moderate in cost, weight, and size compared to the one used for SRM, but extensive control systems are required. Ross et al. postulated that dry reforming should be considered the most viable process amongst the four, since it has 20% lower running costs compared to the other processes, although this statement raises a lot of controversy due to the maturity level of DRM in terms of industrialization [40,48]. Furthermore, it reduces the overall greenhouse gas emissions (carbon dioxide and methane) by using them as reagents for the catalytic process [40]. This is particularly true in the case of design modifications of SRM technology, which can make it more economically viable, e.g., storage and utilization, carbon capture, and solar concentrators [49]. Additionally, it has a H_2_:CO ratio of 1:1, which is ideal for selecting long-chain hydrocarbons in FT [23,50,51,52,53,54,55,56]. 

**Figure 5:**
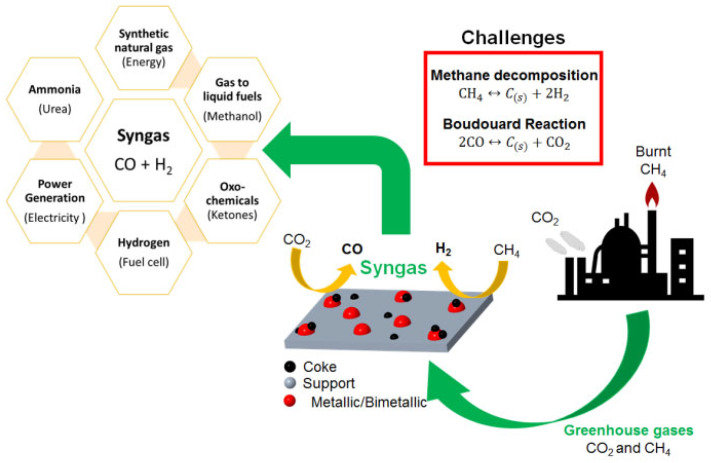
A schematic illustration of the catalytic dry reforming of methane (DRM) reaction, with carbon dioxide as the oxidant, and the use of syngas.

The produced syngas via the endothermic catalytic DRM reaction can be converted into more valuable hydrocarbons, such as methanol, or used as liquid synthetic motor fuels (e.g., diesel, gasoline, kerosene, and naphtha) [6,57]. Syngas can also be used to transport and store solar energy in the form of chemical energy [58,59]. The schematic illustration shown in Figure 5 summarizes the uses of syngas. Additionally, DRM can be used to convert biogas, which has high feedstocks of CO_2_ and CH_4_, along with CO_2_-rich natural gas, which is not considered as valuable as other hydrocarbons, and is usually reformed in refineries into syngas for heating, transportation, electricity production, and industrial purposes [60,61].

## 3. Challenges in DRM Technology

**Figure 6:**
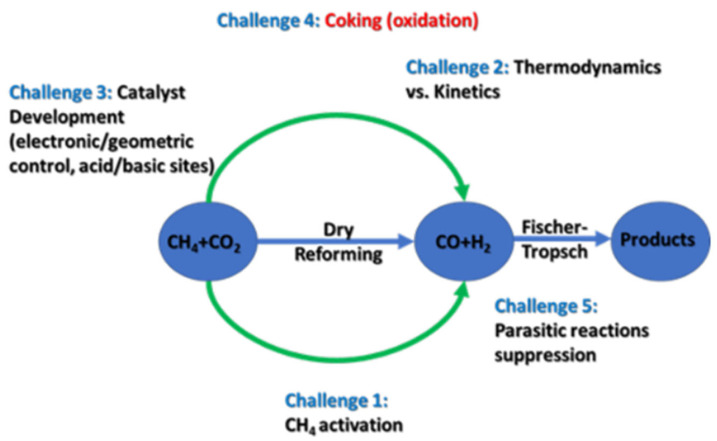
Challenges in the dry reforming of methane reaction.

The major challenges in DRM are discussed in this section, and schematically summarized in Figure 6. In particular, aspects of the reaction—such as metal sintering, support effects, types of carbonaceous species formed, carbonaceous deactivation (i.e., coking), and investigation of DRM deactivation pathways—are all critical to understanding the effort that has been made in recent years to synthesize an economical and stable catalyst for this reaction.

In the following text, important structural parameters, along with their impact on sintering and coking, are discussed. 

### 3.1. Metal Sintering

**Figure 7:**
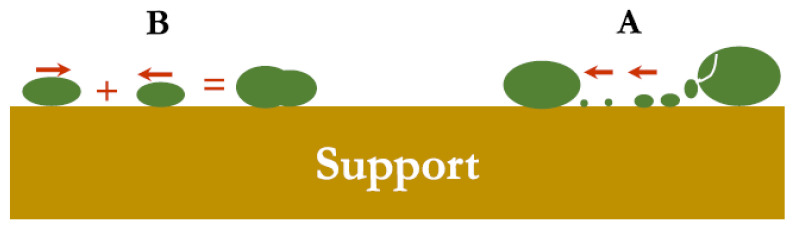
Sintering pathways of catalysts via crystal growth by (A) atomic migration and (B) crystallite migration. Reprinted with permission from Ref. [30]. Copyright 2019 Elsevier.

Metal sintering is a thermally induced irreversible phenomenon (Figure 7) that occurs at high temperatures (≥700 °C), resulting in catalyst deactivation due to surface area reduction through metal growth and pore network collapse. Due to its nature, it is difficult to recycle or regenerate the catalyst; hence, it is crucial to prevent metal sintering, while understanding the fundamentals behind the phenomenon. Under the specific conditions of DRM, metal sintering is stimulated by the presence of moisture from the RWGS side reaction (part of the reaction network during DRM). Active metal growth occurs via two main routes: (i) *atomic migration* by Ostwald ripening and coarsening, and (ii) *crystallite migration* by coalescence. 

In *atomic migration*, the metal atoms are separated from the crystallite and move along the support until they attach to a larger crystallite [62]. Meanwhile, in the case of *crystallite migration*, the entire crystallite moves across the support and forms larger metal agglomerations via collisions and coalescence. These two mechanisms can occur simultaneously via the following physiochemical steps: Firstly, the metal atoms are detached from their crystallite, followed by their entrapment in the pores and surface of the support. Afterwards, the entrapped metal atoms diffuse through the pores of the support. The metal particles cause the wetting of the pores and surfaces, resulting in the nucleation of the metal particles. Finally, agglomerates are formed via coalescence and the attachment of metal particles to larger metal particles. This is an important aspect in the design of a lifetime DRM catalyst to devise strategies for anchoring the metal sites on the support, targeting the suppression of their mobility.

### 3.2. Coking

#### 3.2.1. Metal Sites and Their Impact on Coking Formation

Pakhare et al. discussed the different noble metals used in the development of DRM catalysts [15]. It was mentioned that an inversely proportional correlation exists between the dispersion of the active metal effects and the size of the active site ensembles. Various factors affect the dispersion of the active metal, such as the support, concentration (loading) of metal, promoter addition, type (nature) of metal, and preparation method followed [15]. A higher metal dispersion leads to less carbon formation, while supports with higher surface area (m^2^/g) have been found to definitely contribute to an improvement of the metal dispersion [15]. Oemar et al. reported on the tuning of the particle size and crystal size of the support (Y_2_O_3_) in a Pd–Ni-based catalyst by varying the pH level of the synthesis solution while synthesizing the support via a homogeneous precipitation method [63]. A decrease in the pH led to a decrease in the particle size, which ultimately affected the oxygen mobility in the Y_2_O_3_ lattice; the latter was investigated via temperature-programmed reduction (TPR). Consequently, the enhancement of oxygen mobility resulted in a decrease in the carbon deposition rate. The study showed the absence of NiO and PdO peaks, corroborating the high dispersion of active metals. HR-TEM revealed that the average metal size of finely dispersed Ni-Pd metals was in the 7.0–7.5 nm range. The largest metal particle size was found in Pd-Ni/Y3 (Y_2_O_3_ at pH 3). However, the least carbon formation was exhibited by this catalyst amongst the other three catalysts. Moreover, the discussion of possible methods used to develop carbon-resistant Ni-based systems for dry and steam reforming of methane is of high interest [63]. Many studies have been dedicated to Ni-based catalysts because of their promising potential in terms of chemistry and cost. However, one of the drawbacks of Ni chemistry is its vulnerability to carbon deposition, unlike noble metals, which are more stable and resistant to coking, but also more expensive. As a result, *bimetallic* systems have emerged as a new category of potential metal catalysts to provide a synergistic effect and enhance the ultimate catalyst performance—primarily through ensemble size control; alternatively, noble metals can be used.

**Figure 8:**
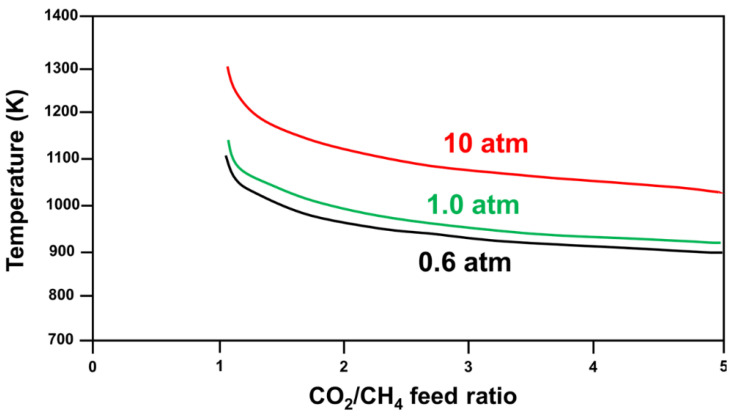
Thermodynamic analysis of total pressures, temperatures, and feed ratios for carbon formation. Reprinted with permission from Ref. [6]. Copyright 2016 Royal Society of Chemistry.

Pakhare et al. reported on Rh-substituted pyrochlores for DRM, and concluded that the temperature range at which DRM should be conducted is 643–1027 °C at atmospheric pressure, as shown in the thermodynamics analysis of total pressures in Figure 8 [64], where each line represents a total pressure at which no carbon formation occurs at a specific temperature and CO_2_/CH_4_ feed ratio [64].

Nikoo et al. reported on some thermodynamic aspects of coking; the authors performed an extensive equilibrium study on DRM, taking into consideration the 16 side (parasitic) reactions that are largely affected by the operating parameters [21], using the thermodynamics principles (−RT ln(K) = ΔG). Hence, reactions with high ln(K) occur throughout the entire temperature range of DRM [21]. As mentioned previously, methane decomposition and Boudouard reactions are the leading causes of carbon formation in DRM. Hydrogenation of CO_2_ and CO, which are exothermic reactions, also cause carbon formation (CO_2_ + 2H_2_ ↔ 4C + 2H_2_O and CO + H_2_ ↔ C + 2H_2_O). Hence, more carbon is formed at low temperatures. At a constant temperature, carbon formation decreases with increasing CO_2_/CH_4_ ratio (>1). Decreasing CH_4_ decreases the source of H_2_, which is the limiting reactant for hydrogenation of CO_2_ and CO; consequently, a decrease in carbon formation is noticed [21].

#### 3.2.2. Support Effect

**Figure 9:**
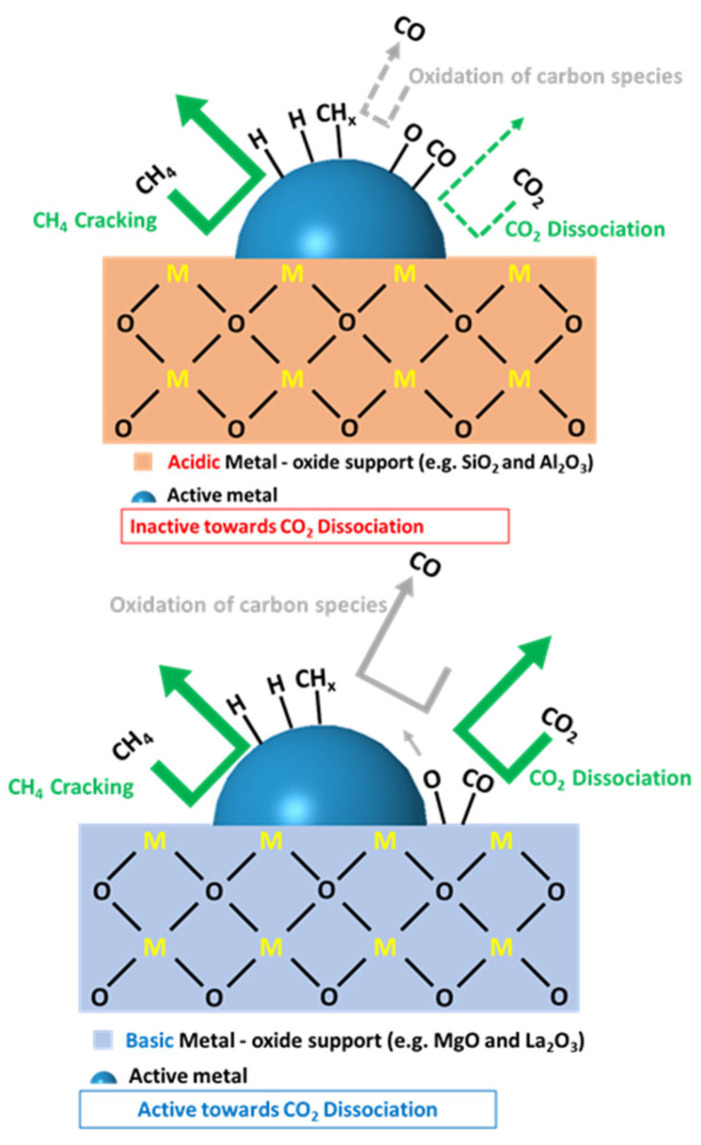
Deactivation mechanisms over Ni supported on acidic and basic metal-oxide supports.

Due to the bifunctional nature of the reaction, the support plays a critical role in the reaction viability as well. According to the literature, the bifunctionality dictates that CO_2_ is activated on the support and CH_4_ is activated on the metal sites. Studies on the physicochemical characteristics of the support showed that the acidity or basicity of the support has an effect on the coke formation route (i.e., via Boudouard or methane decomposition reaction). Basic supports, such as MgO and La_2_O_3_, tend to favor dissociative adsorption of CO_2_, leading to a decrease in the coke formation, as it creates oxygen atoms near the active metal. On the other hand, acidic supports, such as SiO_2_ and Al_2_O_3_, favor the dissociation of methane, thus promoting methane cracking reaction over Boudouard reaction, and leading to coke formation on the surface of the catalyst [65,66,67,68]. Figure 9 illustrates the deactivation mechanisms over a Ni-supported catalyst on acidic and basic metal-oxide supports [69]. Das et al. [69] devised this scheme based on the activity measurements performed on various surfaces with different acidity–basicity levels. It was surmised that Step 1, which represents the methane activation on metallic active sites, is the most important step in the reaction mechanism. Then, methane successively dehydrogenates (Step 2) [70], while the activation of acidic carbon atoms in CO_2_ occurs on surface basic sites (Step 3) and forms MgO-CO_2_, for example. The CO_2_ in MgO-CO_2_ is then transferred to ceria to form ceria oxycarbonates via additive transfer (Step 4). In Step 5, the hydrogen produced via cracking of methane reduces the oxycarbonate formed, contributing to H_2_O and CO production. The produced H_2_O oxidizes the carbon formed and simultaneously produces CO and H_2_. When using an acidic support, methane decomposition leads to the deactivation of the catalyst, because methane decomposition predominates other reactions. Meanwhile, in the case of basic supports, the CO_2_ adsorption is enhanced due to the acidic nature of the CO_2_ molecule [30]. This increases the CO_2_ surface coverage and decreases carbon deposition through the Boudouard reaction [71]. Another aspect of the support’s influence on the ultimate performance is its role in metal crystallite size and dispersion.

#### 3.2.3. Types of Carbon Formed

Studies have shown that the carbon types formed on the catalyst during DRM include a wide spectrum of carbon nanotube (CNT) morphologies, such as amorphous and shell-type carbon, filamentous carbon, polymeric carbon, and graphene [72,73], thus affecting their reactivity and how clean the catalytic surface can be kept. In particular, carbon species with amorphous morphologies (C_α_) tend to be the most reactive (oxidize at c.a. 100 °C); hence, they are easy to gasify [72]. It is known that the oxidation temperature of polymeric carbon species increases with the decrease in the H:C (y/x) ratio in their C_x_H_y_ structure, and this has been proven in many reforming studies for different probe molecules [74,75]. These two types of carbon morphologies are usually referred to as ‘soft’-type carbon, as they do not block active sites. On the other hand, ‘hard’-type carbon—such as graphitic and filamentous carbon species—has much higher oxidation temperatures, making its departure from the surface a real challenge. These are the types of carbons that block active sites and deactivate the catalyst [15]. 

**Figure 10:**
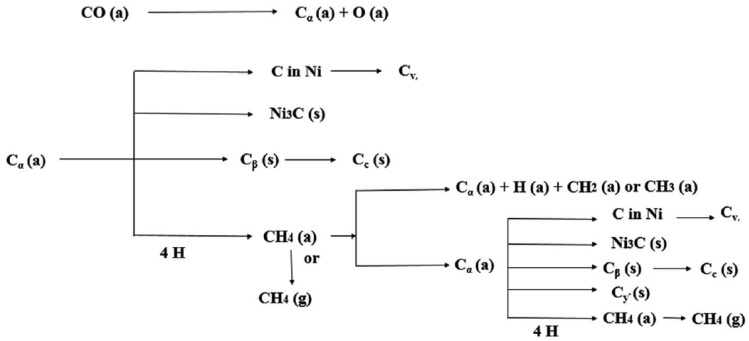
Mechanism of carbon formation at the catalyst surface. Reprinted with permission from Ref. [6]. Copyright 2016 Royal Society of Chemistry.

Arora et al. also classified the carbon formed during DRM into five categories according to their respective oxidation temperature ranges [6], namely, (i) adsorbed or atomic carbon (**C_α_**), (ii) amorphous films or polymers (**C_β_**), (iii) bulk Ni carbide (**C_γ_**), (iv) vermicular filaments or whiskers (**C_ν_**), and (v) graphitic crystalline films (**C_c_**). These carbonaceous species are formed at the following temperature ranges: 200–400 °C, 250–500 °C, 150–250 °C, 300–1000 °C, and 500–550 °C, respectively. In addition, the various types of carbon formed on the surface of Ni-based catalysts, along with the routes they take (i.e., via methane decomposition or Boudouard reaction), are schematically given in Figure 10. 

#### 3.2.4. Characterization of Coking

**Figure 11:**
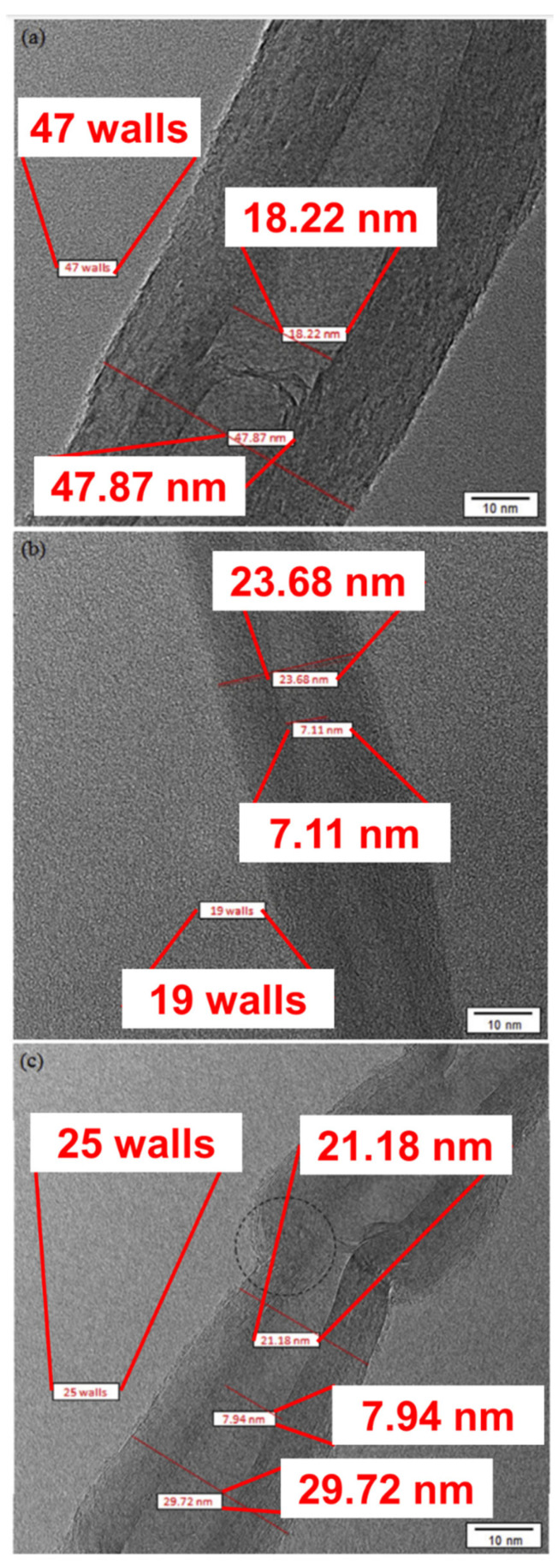
HR-TEM images of the various MWNT allotropes formed on (**a**) Ni/Zr, (**b**) Ni/LaZr, and (**c**) Ni/CeZr during 50 h DRM at 750 °C and constant WHSV = 40,000 mL g^−1^ h^−1^. Reprinted with permission from Ref. [76]. Copyright 2018 Elsevier.

**Figure 12:**
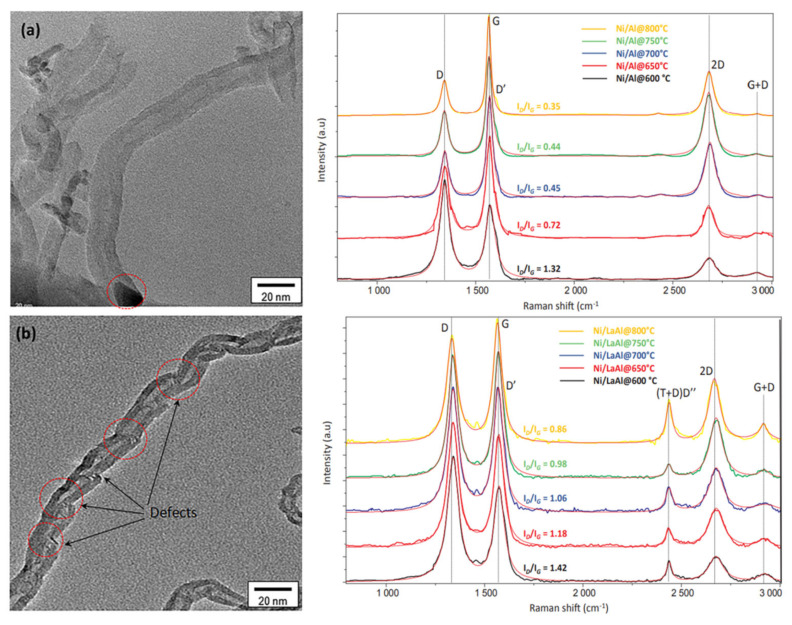
HR-TEM images of spent Ni/Al (**a**) and Ni/LaAl (**b**) catalysts, and their Raman spectra 75. Reprinted with permission from Ref. [77]. Copyright 2019 Elsevier.

Particular techniques are used in order to obtain deep insights into the (micro)structure, morphology, and defects of the carbon entities formed under DRM conditions, such as Raman spectroscopy and transmission electron microscopy (TEM). Charisiou et al. examined the multiwalled carbon nanotubes (MWCNTs) formed on Ni catalysts supported on supports such as ZrO_2_, La_2_O_3_−ZrO_2_, and CeO_2_-ZrO_2_, via HR−TEM, as illustrated in Figure 11 [76]. The same group studied the carbon formed on Ni/Al_2_O_3_ and Ni/La_2_O_3_-Al_2_O_3_ spent catalysts (800 °C) using Raman spectroscopy and HR-TEM, as illustrated in Figure 12 [77]. The HR-TEM depicts the multiwalled carbon nanotubes with defects (shown in red dashed circles) corresponding to the oxygen transferred to the carbon nanotubes (CNTs) during their growth. As a result, the CNTs cannot form continuous and straight walls. The generated defects act as favorable sites for oxidizing gases, consequently making them easily combusted during the catalytic reaction. The Raman D- and G-bands (I_D_/I_G_), appearing at 1300–1400 cm^−1^ and 1500–1600 cm^−1^, respectively, can be used as descriptors for the degree of crystallinity of the CNTs (the higher the crystallinity, the lower the I_D_/I_G_ ratio); the D-band represents the imperfections in polycrystalline graphite, while the G-band represents the out-of-phase intra-layer displacement in the graphene, and is connected to the E_2g_ phonon mode.

The high operating temperatures of DRM, needed for the reaction to be thermodynamically feasible, are accompanied by side reactions that result in carbon formation [13,65] and sintering of the metal-supported catalysts or formation of inactive phases, such as inactive spinels (e.g., NiAl_2_O_4_ in the case of Ni/Al_2_O_3_ catalysts), leading inevitability to its deactivation [14]. Generally, to design a thermally stable, highly active, coke-resistant, and economically feasible catalyst for DRM, a few factors should be taken into consideration that affect the intrinsic properties of the catalysts, such as the interaction between the support and the active metal, the structure of the catalyst, the preparation method, the promoters, the nature of the support, the active metal particle size, and the surface area [68,78]. The pre-treatment reduction temperature can also affect the activity and stability of the catalyst, because it can ultimately affect the metal particle size and the dispersion of active metal sites [79,80].

#### 3.2.5. SSITKA-DRIFTS as a Tool to Track the Coking Pathways

Steady-state isotopic transient kinetic analysis (SSITKA) has been used extensively by researchers to investigate catalytic properties and performance, as well as reaction kinetics, as discussed in a review by Ledesma et al. [81]. Reaction kinetics and mechanisms can be investigated when this technique is combined with mass spectroscopy in transient mode.

In the DRM reaction, the two main pathways of carbon formation—namely, CH_4_ decomposition and Boudouard reaction—as well as their contribution to the total carbon accumulation, can be probed using transient isothermal isotopic experiments (TIIEs) and steady-state isotopic transient kinetic analysis (SSITKA). In particular, SSITKA provides important information about heterogeneous catalysis at a molecular level, and sheds light on the chemical composition of active intermediate species and spectator species using a continuous stirred-tank reactor (CSTR) or a plug-flow reactor (PFR) [82]. It can be used to find the site reactivity and surface coverage (θ) of reaction intermediates that take place in the mechanistic reaction pathways [83]. The combination of steady-state conditions and transient techniques in SSITKA enables the user to calculate other catalytic parameters, such as catalytic performance, activation energy (E_a_), reaction order, [84,85,86] concentration (mol/g) of active reaction intermediates, surface residence time, and intrinsic turnover frequency of the reaction (TOF_ITK_), which is based on the active reaction intermediates [82]. Accurate mechanistic studies can be performed via SSITKA in isobaric (i.e., constant pressure) and isothermal (i.e., constant temperature) conditions and with a constant overall composition of the reaction, which means that the composition of the adsorbed phase on the surface of the catalyst does not change. Tsipouriari et al. used isotopically labelled molecules (i.e., ^13^CO_2_, ^13^CH_4_, and C^18^O_2_ isotopes) to trace the origins of carbonaceous species formed on the surface of Ni/La_2_O_3_ and Ni/Al_2_O_3_. It was found that the La_2_O_3_ support had a higher activation rate of CO_2_, which resulted in the formation of La_2_O_2_CO_3_; the latter decreased the carbon formation [68]. Szedlacsek et al. [87] mathematically modelled SSITKA to obtain the intrinsic rates of adsorption and desorption of species in the reaction, the equilibrium exchange rates of the adsorbate between the gas and surface phases, and the rate of desorption in terms of θ—information that was used to determine the intrinsic characteristics of the catalytic surface. 

Efstathiou et al. used SSITKA and TIIE extensively to understand the origin of the carbon as well as the lattice oxygen mobility in the ceria-based Ni catalysts [6,17,30,55,64,88,89,90]. The authors concluded that methane decomposition is generally the main source of carbon formation. Vasiliades et al. used ^18^O_2_ TIIE to probe the extent of lattice oxygen participation in gasifying the carbon formed via carbon pathways (i.e., CH_4_ decomposition and CO dissociation reaction) during DRM over Ni-Pt/Ce_0.8_Pr_0.2_O_2−δ_ [91] and NiCo/Ce_0.75_Zr_0.25_O_2−δ_ [89] catalysts_._ It was found that CH_4_ decomposition is the main source of inactive carbon formation over Ni and Ni-Pt catalysts supported on Ce_0.8_Pr_0.2_O_2−δ_ [91]. However, in the case of Pt supported on a Ce_0.8_Pr_0.2_O_2−δ_ catalyst, CO disproportionation reaction was the main source of carbon formation [91]. Additionally, the NiPt bimetallic catalyst showed higher labile oxygen participation. Similarly, Damaskinos et al. used isotopic oxygen (^18^O_2_) to investigate the extent of oxygen lattice participation in the gasification of carbon formed via the main carbon routes in DRM over X% Ni/Ce_0.8_Ti_0.2_O_2−δ_ (X = 3%, 7.5%, and 10%) [90]. In the same study, isotopically labelled ^13^C carbon dioxide (^13^CO_2_) was used to distinguish the source of the carbon formed during DRM. The study concluded that CH_4_ decomposition is the main source of inactive carbon formation. 

#### 3.2.6. Regeneration of Spent Catalyst

The inevitable deactivation of catalyst during DRM due to sintering of metal active sites and carbon formation makes it imperative to find an economically and environmentally friendly solution to discard the inactive catalyst. Regeneration provides a practical solution, but it depends on the reversibility of the source of deactivation [29,92]. Catalyst deactivation can occur by fouling (i.e., blocking the active sites on the surface of the catalyst, e.g., coke formation), poisoning (i.e., feed or product species strongly chemisorbed on the active sites), thermal degradation, or sintering [93,94]. Regeneration is not always possible or economically feasible. For instance, metal sintering is considered difficult or irreversible, with the exception of some noble metals. On the other hand, coking can be easily reversed by oxidizing the carbon formed (gasification) [3,62,95]. Gasification can be performed by temperature-programmed oxidation (TPO) at an increased temperature (300–500 °C) under an oxidant stream, such as CO_2_ [31], O_2_ [96], air [97,98], steam [99], or hydrogasification [100]. Gasifying at low temperatures restores the catalyst without destroying its pore structure or composition. Additionally, the method of regeneration depends on the (i) design, (ii) type, and (iii) configuration of the catalyst reactor. For instance, in the case of fixed-bed reactors, ex situ regeneration is possible after a cycle of reaction. However, in situ regeneration is performed when using fast-deactivating catalysts, and in a system that allows the oxidation of the catalyst via CO_2_ along with the DRM reaction [30].

Theofanidis et al. [31] studied the removal of carbon on the surface of an 8 wt.% Ni–5 wt.% Fe catalyst supported on a MgAl_2_O_4_ support via CO_2_ and O_2_ as oxidants. Operando time-resolved XRD, temperature-programmed oxidation (TPO), and isothermal temporal analysis of products (TAP) experiments were performed to mechanistically investigate the carbon removal. The results showed that CO_2_ was able to remove the carbon on the metal active sites. However, the EDX-STEM mapping performed showed that the presence of carbon species was away from the metal active sites. This means that there was no direct interaction between the carbon species and the CO_2_ gas passed over the catalyst. The mechanism followed by CO_2_ is as follows: Initially, CO_2_ dissociates on Ni metal particles and, subsequently, the carbon oxidizes via surface oxygen. Simultaneously, CO_2_ oxidizes Fe into iron oxide, and then the Fe oxide is reduced and donates its oxygen to oxidize the carbonaceous species. In the case of O_2_, the oxidation of Fe and Ni (Fe_2_O_3_ and NiO) occurs first. Then, mobile lattice oxygen (O_L_) from the active metal oxides gasifies the surface carbon. The oxidized particles migrate on the surface of the catalyst and oxidize the carbon species that they come in contact with. Additionally, O_2_ spillover has a negligible contribution to carbon gasification. Wu et al. were able to remove carbon from a Rh_0.1_Ni_10_/BN catalyst at 300 °C and activate the catalyst, but a small decrease in the activity during DRM was observed after regeneration [101]. Vasconcelos et al. [97] studied the regeneration ability of a spent nickel-hydroxyapatite-based catalyst after DRM (20% of CH_4_/20% of CO_2_/60% of N_2_ at 700 °C for 30 h) using two different gasifying agents, namely, air and CO_2_ (20% CO_2_/N_2_). The catalysts exhibited 80–90% conversion rates and no change in selectivity after three successive cycles of DRM/regeneration. Some irreversible deactivation was due to the formation of core–shell structures between coke and Ni.

## 4. Learning-Driven Design of DRM Catalysts

### 4.1. Noble Metals: Their Role in Controlling the Coking

Noble metals such as Pt, Rh, Pd, Ir, and Ru are highly active in DRM, thermally stable, and more coke-resistant than transition metals, but they are expensive [102,103,104,105]. Intuitively, the addition of noble metals to Ni decreases coking. Consequently, in an approach to make the catalysts financially feasible, noble metals can be incorporated into transition metals—such as Ni- and Co-based catalysts—at small loadings (up to 0.5%) as solute elements. Studies on Ni–noble-metal-supported systems such as Ni, Pt, and Ru on ZnLaAlO_4_ showed that the addition of Ru and Pt increased the activity of the catalyst. TGA results showed that the carbon formation decreased dramatically in the presence of Pt and Ru compared to monometallic Ni [106]. Studies performed by Foppa et al. showed that the carbon formation on noble metals such as Pt and Pd is predominantly due to methane cracking, while coke formation on Ni is due to CO disproportionation reaction [107]. As mentioned in the previous section, the carbon species formed from methane cracking can be easily gasified using carbon dioxide as an oxidant medium. This is the main reason behind noble metals’ high coke resistance. However, their use is limited due to their cost.

Ferreira-Aparicio et al., examined the performance of Ni, Co, Pt, Rh, and Ir active sites supported on Al_2_O_3_ and SiO_2_ for the DRM reaction. Their study showed that catalysts with Ru and Rh metal sites had the highest catalytic activity and stability [108]. This was theoretically confirmed via first-principles calculations, which showed that Ru and Rh have higher activity than Ni, Pd, and Pt metal active sites with the same dispersion and particle size [109]. In general, many studies have reported on the superiority of Ni-promoted catalysts with noble metals (e.g., Pt, Rh, Ru, or Pd) in terms of their higher activity and coke resistance compared to the non-promoted Ni catalysts [110,111,112,113]. For instance, Menegazzo et al. reported that a Ni-Pt/ZrO_2_ bimetallic catalyst showed improved activity and longevity of the catalyst for DRM compared to the monometallic Ni/ZrO_2_ catalyst [114]. While, the presence of Rh with low loading (Rh:Ni = 1:100) in Rh-Ni/Al_2_O_3_ increased the activity and stability of the catalyst compared to its monometallic counterparts [111]. This was attributed to the hydrogen spillover, which increased the reducibility of Ni, therefore keeping Ni in its metallic form (Ni^0^), which consequently maintained the catalytic activity of the catalyst. Similar trends of improved catalytic activity and stability were exhibited over a Co/TiO_2_ catalyst with the presence of Pt (Pt/Co = 0.005–0.05 in atomic ratio) and Ru (Ru/Co = 0.01–0.05) [115]. 

### 4.2. Non-Noble-Metal (Ni-Based) Catalysts

Conversely, the limited availability and high price of the noble metals make it difficult to use these metals as catalysts on an industrial scale for DRM [116,117]. The comparable activity of nickel with that of noble metals has attracted the attention of researchers, and led to hundreds of related publications in just one year [118]. The DRM reaction occurs simultaneously with other side reactions that affect the performance of the catalyst negatively, namely, reverse water–gas shift (RWGS; Equation (2.1)), methane cracking (Equation (2.2)), and Boudouard reaction or CO disproportionation reaction (Equation (2.3)). The temperatures at which these side reactions occur can be determined using the standard free energies (∆G°). Wang et al. deduced from the free energy equations that DRM occurs at temperatures greater than 640 °C, while RWGS occurs below 820 °C. Meanwhile, the carbon deposition occurs in the 557–700 °C temperature range; this is attributed to the fact that the methane decomposition reaction occurs at T > 557 °C and the CO disproportionation reaction occurs at temperatures below 700 °C [17]. The CO disproportionation reaction and methane cracking cause carbon deposition, and eventually lead to the deactivation of the catalyst. The carbon species formed is of great interest, because it affects the regeneration process of the catalyst.

**Figure 13:**
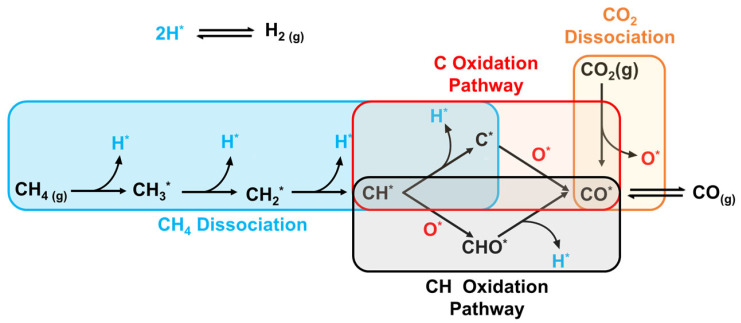
Reaction mechanism of dry reforming of methane (DRM) pathways. Reprinted with permission from Ref. [123]. Copyright 2014 Elsevier.

The nature of the carbon is affected by the reaction from which it resulted (Equation (2.2) or Equation (2.3)). It is evident from the enthalpy change of the Boudouard reaction that it is an exothermic reaction, which means that it favors lower temperatures. Hence, it is inhibited when DRM operates at temperatures greater than 800 °C, and the carbon species formed are mainly from methane cracking. As a result, the carbon deposition on the surface of the catalyst is minimal. This can be attributed to the fact that the carbon species formed by methane cracking are more reactive than the ones produced by the CO disproportionation reaction. This means that the rate of gasification of carbon via carbon dioxide is greater than the rate of carbon formation by methane cracking [119]. Meanwhile, at the operating temperatures of CO disproportionation reaction, a less-active carbon is formed at the surface of the catalyst, and leads to its deactivation [15]. This was experimentally proven by Tomishige et al., who showed that the carbon formed on the surface of a Ni-Mg-O solid solution at high temperatures was mainly due to methane cracking, and it was observed that methane led to the sintering of Ni more than Co [120,121]. Another study showed that at 850 °C most of the carbon was formed on the catalyst’s surface at the inlet of the reactor, proving that the carbon deposition was predominantly due to methane cracking [122]. Carbon formation on Ni-based catalysts was extensively studied to understand the major reasons behind coking. Studies showed that the carbon species from methane cracking and CO disproportionation reactions dissolve into the active metal (Ni) and form nickel(III) carbide [122]. This is a crucial step in carbon formation. Furthermore, the particle size of Ni, the plane of nickel exposed, and the diffusion and segregation of carbon affect the carbon growth. It was shown that Ni(100) and Ni(110) surfaces lead to more coking than Ni(111) surfaces [15]. This is due to the fact that Ni(111) has a high energy barrier for CO disproportionation, and it favors the CH pathway, as illustrated in the reaction mechanism shown in Figure 13 [123].

### 4.3. Other Transition Metal Catalysts

The high price of noble metals has led researchers to explore less expensive alternatives, other than Ni-based catalysts, to implement catalysts on an industrial scale. The intrinsic characteristics of transition metals affect their role in the bimetallic system. Cobalt alone has previously been used for DRM [116,124]. On the other hand, not all supports are active with Co for DRM. It was found that only Co/γAl_2_O_3_ and Co/MgO are active in DRM. The numerous studies performed on Ni-Co bimetallic systems show that the ratio of Co and Ni and the support determines the catalyst’s activity. Zhang et al. prepared a Co-poor catalyst (Ni–Co–Al–Mg–O) via co-precipitation; their catalyst showed great characteristics, such as long life, coke resistance, and greater stability [20]. A recent work showed that adding small amounts of Co enhances the catalytic activity; this can be attributed to the fact that Co has strong affinity for oxygen species [125]. However, it is more prone to poisoning from the surface oxygen [109]. Ni-Cu and Ni-Fe systems have been also investigated extensively, and showed favorable catalytic characteristics in DRM due to coke resistance [126] and excellent redox properties [127], respectively. Consequently, the presence of Fe leads to realloying and dealloying processes, causing fast removal of coke.

### 4.4. Strong Metal–Support Interaction (SMSI)

The interaction between the metal and the support is classified based on the extent of the interaction. This phenomenon is categorized into (i) *weak metal–support interactions* (WMSIs) and (ii) *strong metal–support interactions* (SMSIs) [128]. In 1978, Tauster et al. discovered that the chemisorption of the species decreased when the support was reduced at high temperatures. Consequently, the term strong metal–support interaction (SMSI) was used to describe the electrocatalytically significant interaction between the active metal and the support [79,80,129,130]. Traditionally, SMSI is a thermal catalysis term. However, can be used in electrocatalysis to describe a similar phenomenon (i.e., electron flow through the interface), as mentioned in [131]. Pan et al. also discussed the importance of metal–support interactions in electrocatalysis [128]. Studies showed that SMSI usually appears when using reducible supports such as ceria, titania [58], vanadia [58,132], etc. Originally, SMSI was thought to occur due to the formation of intermediate phases, or due to the electron transfer between the metal and the support [133,134]. Currently, scientists conceptually assume that SMSI phenomena are connected to the interfacial and transport phenomena and charge redistribution during metal–support interface interactions. The SMSIs have electronic, geometric, and bifunctional effects that ultimately affect the selectivity, activity, and stability of the catalyst. As a result, understanding the mechanisms behind SMSI is vital for tuning catalysts with desired properties. These effects are further discussed in the next few sections.

#### 4.4.1. Electronic Effect

**Figure 14:**
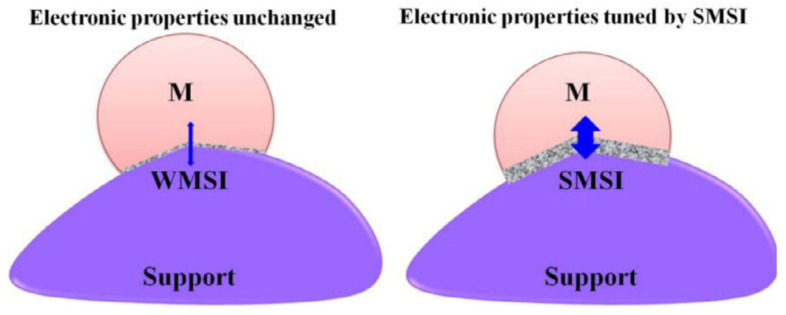
Schematic representation of the main metal–support interactions, namely, weak metal–support interactions (WMSIs) and strong metal–support interactions (SMSIs). Reprinted with permission from Ref. [128]. Copyright 2017 Elsevier.

Works carried out by Solymosi [135] and Schwab [129,136] demonstrated the electronic effects induced by SMSIs. These changes are caused by the redistribution of electrons at the interface. Studies on non-transition-metal-oxide supports (i.e., without d-orbitals), such as MgO supports, revealed that the favored interactions occur with the oxide anion [137]; the latter results in weak interaction between the oxygen sites and the metal ions. These metal oxides do not favor the formation of nucleation centers, due to the ease of migration. On the other hand, transition metal oxides that have d-orbital electrons, such as TiO_2_, result in weak covalent interaction between cations, d-orbital electrons, and the metal atom [138]. Furthermore, dominant interactions occur between reduced metal cations and the adjacent metal atom (ionic). The surface defects on these supports act as electron traps, which consequently increase the electron density of the metal atoms. Figure 14 illustrates the electronic effects caused by WMSI versus SMSI [128].

The interfacial contact that occurs between the metal and the support results in charge redistribution in the interface. The degree of interaction (weak or strong) between the metal and the support is governed by continuity of electric potential and energy minimization [139]. The electronic structure of the metal changes, and new phases are formed (a few nanometers thick) [140] at the reactive interface, in the case of strong metal–support interaction (SMSI). However, the electronic structure of the metal is unchanged in the case of weak metal–support interaction (WMSI). Hence, the nature of the MSI tunes the catalytic performance of the catalyst by controlling the electron transfer [128]. 

The amount of electron transfer depends on the size of the metal clusters and the structure of the semiconducting oxides, such as titania [128]. Ioannides et al. [141] developed a theoretical model based on the metal–semiconductor (MS) contact theory to analyze the electron charge transfer in Group VIII metal clusters supported on doped TiO_2_. The electron density transferred to the metal is inversely proportional to the metal cluster.

Generally, MS theory can be used to describe the contact between metals and semiconducting oxide supports [130,142,143]. The imbalance between the Fermi energy levels of the metal and the support leads to electron transfer between the metal and the support [144]. Ultimately, the electron transfer affects the charge distribution and density of the metal species, affecting the catalytic properties of the catalyst. Hence, the electron-transfer process is particle-size-dependent. 

There are two types of MS contacts: (i) rectifying Schottky contact, and (ii) non-rectifying Ohmic contact. The different types of MS contacts form due to the mismatch in Fermi levels between the metal and the semiconducting oxide, due to a difference in the work function (Φ) [145]. The Schottky barrier contact occurs when the MS contact has a large potential barrier height formed when the Fermi energy levels of the metal and the semiconductor are aligned together. Both n-type and p-type semiconductors can form Schottky contact, such as in the cases of titanium silicide and platinum silicide. Meanwhile, in the case of Ohmic contact, no barrier is formed, and it usually occurs when the work function of the metal (Φ_M_) is less than the work function of the semiconducting support (Φ_S_) [145]. This causes electron transfer from the metal to the semiconducting oxide until the Fermi level in the semiconductor reaches equilibrium [145]. 

#### 4.4.2. Geometric Effect

The geometric effect caused by SMSI involves the decoration of metal clusters either by partial coverage of the metal surface or total coverage of the metal surface (i.e., encapsulation) [146,147,148,149,150,151]. Encapsulation is followed by partial reduction of the oxide due to high temperature. The geometric effect is caused by the metal–support interactions [152]. A two-step encapsulation mechanism is utilized to describe the geometric effect. For example, in the case of titania, initially, Ti^+3^ or Ti^+4^ interstitial cations undergo mass transport towards the surface, which is supported by the high temperature, which promotes the diffusivity of Ti in the titania support. Then, encapsulation reactions take place when the surface energy (γ) of the metal is greater than the surface energy of the support [153]. The driving force of the encapsulation is the minimization of total energy. Hence, SMSI favors supports with low surface energy, such as titania, and metals with high surface energy, such as Pt and Pd [151,154]. Work function is another parameter that affects encapsulation. Metals with low work function favor oxidation reaction at the interface, while metals with high work function favor encapsulation [128].

#### 4.4.3. Bifunctional Effect

**Figure 15:**
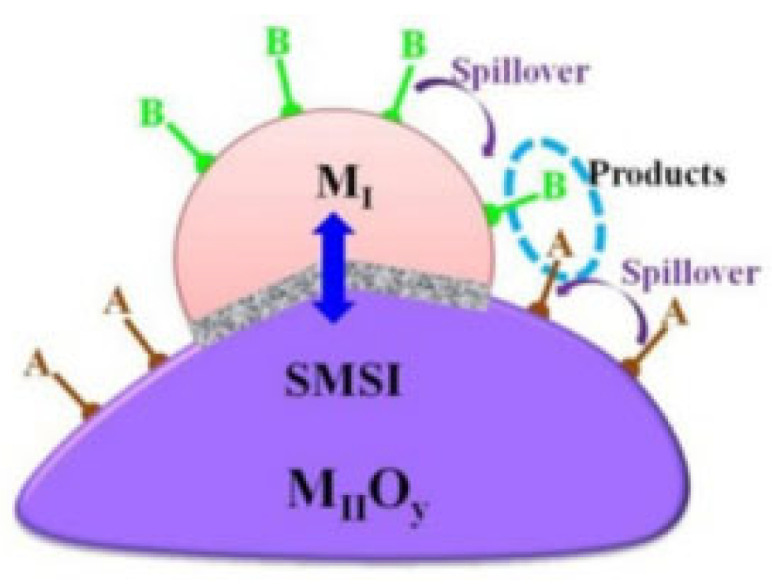
Spillover: an example of the bifunctional effect caused by SMSI. Reprinted with permission from Ref. [128]. Copyright 2017 Elsevier.

In this case, the interaction between the metal and the support provides separate and dual reactive sites. The spillover phenomenon results in increases in selectivity and activity. In this phenomenon, the adsorbed species from the support or the metal move towards the interface and create another reaction site, which is called a dual site [155], as schematically illustrated in Figure 15 [128]. The oxygen vacancies near the interface caused by the reduced layer of the support also participate in the reaction, as they act as dual-function sites [156]. The high concentration of reduced oxide cations improves the activity and selectivity. Additionally, the density of the interface increases due to the SMSI as the catalyst is exposed to high temperatures. This results in the formation of triple-phase sites [157].

The nature of the supports used in the catalytic system plays an important role in the activity of the catalyst in DRM, since the support affects the kinetic and mechanistic steps of DRM, which are also connected to the carbon formation. Numerous studies have examined noble metal (e.g., Pt, Pd, Rh, and Ru) catalysts [157,158,159,160,161] and transition metal catalysts [162,163] loaded on a series of supports (such as SiO_2_, La_2_O_3_, ZrO_2_, TiO_2_, CeO_2_, Al_2_O_3_, and MgO) for use in DRM. DRM follows a bifunctional mechanism on acidic/basic support sites, such that CH_4_ is activated on the active metal site while CO_2_ is activated on the acidic/basic sites of the supports. In the case of acidic supports, CO_2_ is activated by the surface hydroxyl groups, forming formate species. Meanwhile, in the case of basic supports, CO_2_ activation takes place through formation of oxycarbonates [164,165]. On the other hand, DRM follows a monofunctional pathway when the active metals are supported on inert supports. This means that the activation of both reactants (i.e., CO_2_ and CH_4_) during DRM takes place on the metal active site. Initially, carbon is formed via CH_4_ dehydrogenation, and then CO_2_ is activated and reacts with carbon, leading to the deactivation of the catalyst [164,165]. The different pathways followed via inert and acidic/basic supports affect the carbon formation during DRM, as well as the metal–support interactions. Studies have shown that inert supports such as silica (SiO_2_) lead to WMSI, which decreases the activity and stability of the catalyst compared to acidic/basic supports [108,166,167]. WSMI and inert supports are not necessarily considered drawbacks when dealing with bimetallic catalysts, as in the case of a Rh-Ni/boron nitride (BN is an inert support) catalyst, which showed higher conversion rates of CO_2_ and CH_4_, and higher stability, along with lower carbon formation than Rh–Ni/γ-Al_2_O_3_ (γ-Al_2_O_3_, acidic support) [101]. The superior performance exhibited via Rh-Ni/boron nitride is linked to the negligible metal–support interaction, which allows the migration of metal clusters and formation of Rh-Ni clusters (mobility), where the close atomic proximity of Rh metal to Ni metal leads to the decrease in carbon formation without affecting its inherent activity. On the other hand, the SMSI in the Rh–Ni/γ-Al_2_O_3_ catalyst hindered the metal mobility and prevented the formation of Rh–Ni metal clusters [101]. It is noteworthy to mention that the effect of metal mobility on the dispersion of the active metal sites was not addressed in this study [101]. The study performed by Wu et al. [101], concluded that the DRM mechanism is affected by both the active metal and the nature of the support. This was made apparent by replacing Rh metal with Pt metal, which assisted the formation of bimetallic Pt-Ni clusters on the γ-Al_2_O_3_ support, and was accompanied by an increase in the stability and activity of the catalyst for over 100 h [168].

### 4.5. Metal Dispersion and Carbon Formation

There are a lot of studies on the relationship between carbon formation and the size and loading of the Ni active metal. Crnivec et al. studied the effects of the metal active site and loading on the catalytic performance of Ni–Co/CeO_2_- ZrO_2_ catalysts for DRM [169]. The Ni–Co-based catalysts had different metal loadings, specifically, 3, 6, 12, and 18%. As the metal loading increased, the particle size of Ni-Co increased, resulting in higher carbon accumulation during DRM. This means that SMSI phenomena are dominant at low metal loadings. Additionally, catalysts with small metal particle size (<6 nm) had better carbon resistance compared to their counterparts, while exhibiting selectivity towards the RWGS reaction. However, larger metal particles promoted methane decomposition and, consequently, increased coking. Wang et al. reviewed numerous catalytic systems for DRM studies to design a carbon-free catalyst. It was reported that CH_4_ conversion was related to the metal loading—the higher the noble metal loading, the higher the conversion [17]. Low noble metal loadings were proven to give sufficient performance during DRM. However, higher metal loadings are required when using transition metals such as Ni and Co [17]. Another study performed by Gohier et al. studied the effect of Ni particle size on the growth of carbon nanotubes (CNTs) [170]. Metal particles with crystallite size below 5 nm resulted in the growth of single-walled (SWNT) or few-walled CNTs, and the growth occurred outwards (i.e., CNTs grew above the metal seed). However, large crystallite size—greater than 15 nm—resulted in multiwalled CNTs (MWNTs), and the growth occurred inwards (i.e., CNTs grew below the metal seed).

Studies have shown that large Ni particles increase carbon formation during DRM [171,172,173]. The high operating temperature of DRM leads to the sintering of the initially small Ni particles, and eventually increases carbon formation. However, SMSI between the Ni particles and supports—such as Al_2_O_3_, CeO_2_, and MgO—can eventually lead to small Ni particles and/or maintain the size of Ni during high reaction temperatures of DRM. This can happen either through surrounding/decorating the Ni phase with oxide/suboxide entities, or through the formation of new Ni-supported phases (e.g., NiAl_2_O_4_). Nonetheless, long reaction times inevitably lead to the enlargement of the Ni particle size [71,118,174,175,176,177,178,179,180,181,182]. The size effect is coupled with the support effect, so it is difficult to attribute the enhancement in performance and preclusion of coking to the size effect or the support effect [183,184,185].

**Figure 16:**
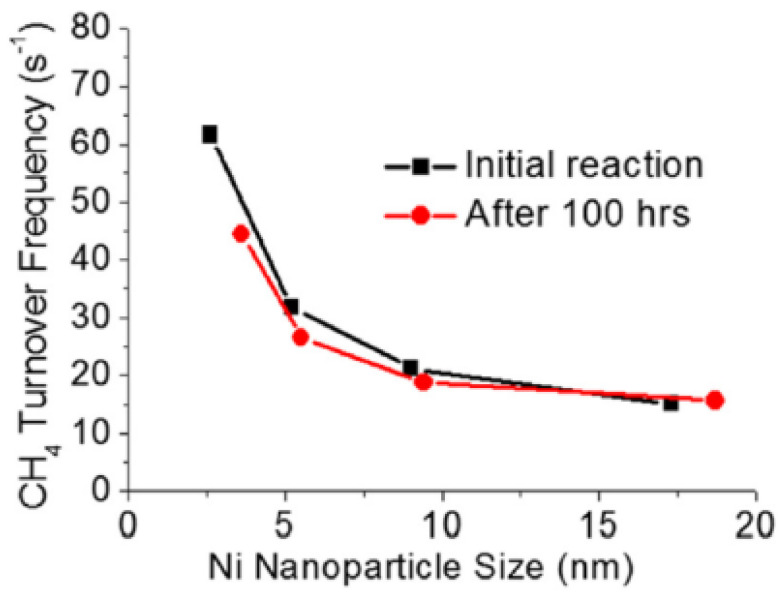
Effect of Ni nanoparticle size on CH_4_ turnover frequency. Reprinted with permission from Ref. [186]. Copyright 2017 Elsevier.

Hence, examining the metal size and support effects independently can provide researchers with deeper insight. Han et al. were able to synthesize a unique catalyst to study each effect (size and support) separately [186]. A range of nano-sized Ni particles (2.6, 5.2, 9.0, and 17.3 nm) were first immobilized on silica spheres, and then the Ni/SiO_2_ catalyst was coated with a metal oxide overlayer, such as SiO_2_, TiO_2_, Al_2_O_3_, ZrO_2_, or MgO. The effect of Ni size on the DRM reaction for 100 h at 800 °C was evaluated based on the turnover frequency (TOF), which estimates the activity of the catalyst per Ni surface atom per time; the TOF and the conversion rates of CH_4_ and CO_2_ were higher in smaller Ni particles on the Ni/SiO_2_@SiO_2_ catalysts. In particular, the 2.6 nm Ni particles exhibited fourfold higher TOF than the 17.3 nm Ni particles, as illustrated in Figure 16. This significant increase in TOF can be explained by the increase in the surface fraction of edges and vertices of 2.6 nm Ni nanoparticles (6.5 times higher), which were calculated via the Van Hardeveld and Hartog method [187]. Additionally, the CO-TPD performed before and after DRM, used to measure the available Ni sites, showed that the CO uptake increased after DRM, which was attributed to the high-temperature reaction that led to the increase in the pore size of the silica overlayer. Moreover, TEM, TPO, and Raman results indicated that the silica overlayer inhibited carbon formation, such that no filamentous carbon, no peaks at higher than 400 °C, and no D- or G-bands were observed, respectively. However, the absence of the silica overlayer increased the size of the Ni particles via aggregation, and resulted in carbon formation.

## 5. Support Types for DRM Reaction

### 5.1. Metal Oxide Supports: Intrinsic Characteristics

Traditionally, supports are used to disperse active metal sites, although there are an increasing number of studies highlighting the important role of the support in the performance of the catalyst and carbon resistance [179]. The textural and physicochemical properties of a support affect the dispersion of the active metal and, ultimately, the catalyst’s carbon inhibition. Additionally, the interaction between the support and the active metal species affects the catalyst’s reducibility and stability. Hence, it is of paramount importance to select an appropriate support with desirable characteristics, such as surface area and pore volume, redox properties, oxygen storage capacity (OSC), surface basicity, and thermal stability [188]. Exploiting these chemical and textural properties of a support can enhance the metal–support interaction (MSI), improve the dispersion of active metal sites and, consequently, prevent their sintering during DRM, which may result in reduced carbonaceous species formation. 

#### Labile Oxygen Species

The metal oxides can be roughly categorized into reducible and irreducible types. In principle, the descriptor for the reducibility of an oxide is the energy of formation of a vacancy (O_v_). The reducibility (mobile oxygen species) can be tuned by doping and/or nanostructuring [189]. The lability of lattice oxygen plays a predominant role in many reactions, including WGS, DRM, reforming, and oxidation [6]. The role of labile oxygen in the reactions is witnessed in a plethora of cases, as discussed below. Clear demonstration of the above was given by Wang and Ruckenstein [190], where the performance of Rh-based (0.5 wt.%) catalysts supported on reducible and irreducible catalysts during DRM under the same reaction conditions was investigated. The conversion rates of CH_4_ and CO_2_ reflect the influence and importance of the nature of the support on the activity and stability of the catalyst. The study concluded that the irreducible metal oxide supports—namely, SiO_2_, γ-Al_2_O_3_, MgO, La_2_O_3_, and Y_2_O_3_—generally had higher conversion rates and H_2_/CO ratios than the reducible metal oxide supports (TiO_2_, Nb_2_O_5_, Ta_2_O_5_, CeO_2_, and ZrO_2_). After 30 min of DRM (CH_4_/CO_2_ = 1, T = 800 °C), the Rh-based (0.5 wt.%) catalysts supported on irreducible metal oxides exhibited a decrease in the conversion rates of reactants (CH_4_ and CO_2_) and yields of CO and H_2_. The 0.5 wt.% Rh/La_2_O_3_ exhibited the lowest conversion rates, followed by the 0.5 wt.% Rh/Y_2_O_3_ catalyst. Meanwhile, Rh-based catalysts supported on γ-Al_2_O_3_, SiO_2_, and MgO metal oxides showed a similar conversion rates and CO and H_2_ yields. However, when the time on stream (TOS) was increased to 50 h, a drastic deactivation of 0.5 wt.% Rh/SiO_2_ was observed, and the trend was changed as follows: γ-Al_2_O_3_ ≈ MgO > Y_2_O_3_ > La_2_O_3_ ≈ SiO_2_. It is noteworthy to mention that the 0.5 wt.% Rh/Y_2_O_3_ catalyst exhibited deactivation after the TOS was increased to 50 h. In the case of reducible supports, the conversion rates and CO and H_2_ yields followed the order Ta_2_O_5_ >TiO_2_ > ZrO_2_ > Nb_2_O_5_ > CeO_2_ after 30 min of DRM (CH_4_/CO_2_ = 1, T = 800 °C). When the TOS was increased to 50 h, the 0.5 wt.% Rh/Ta_2_O_5_ catalyst suffered from deactivation. The deactivation of the 0.5 wt.% Rh/SiO_2_, 0.5 wt.% Rh/Y_2_O_3_, and 0.5 wt.% Rh/Ta_2_O_5_ catalysts was attributed to the metal sintering. On the other hand, γ-Al_2_O_3_, MgO, and La_2_O_3_ exhibited high stability, which was related to the strong metal–support interaction (SMSI). In the case of MgO and La_2_O_3_ supports, LaRhO_3_ and MgRh_2_O_4_ compounds were found to be formed. 

Similarly, Nakagawa et al. [191] studied the activity of Ir-based catalysts supported on ZrO_2_, Al_2_O_3_, Y_2_O_3_, La_2_O_3_, MgO, SiO_2_, and TiO_2_. The study showed that only Al_2_O_3_ and SiO_2_ exhibited carbon formation and, ultimately, led to the low stability of the catalysts. It is noteworthy to mention that the TiO_2_ catalyst had the highest activity amongst the catalysts. The support activity order towards DRM with iridium catalysts was found to follow the order TiO_2_ > ZrO_2_ > Y_2_O_3_ > La_2_O_3_ > MgO > Al_2_O_3_ > SiO_2_.

Zhang et al. [192] investigated the effects of supports on Ni-based catalysts’ performance in DRM. Six supports—namely, ZrO_2_, TiO_2_, MgO, SiO_2_, Al_2_O_3_, and MgO-modified Al_2_O_3_ (MA) supports—were studied. The 8 wt.% Ni/SiO_2_ showed a low carbon formation rate of 3.0 mgC g_cat_^−1^ h^−1^ after DRM (CH_4_/CO_2_ = 1, T = 750 °C), and the highest conversion rates due to its high surface area (235 m^2^/g) and metal dispersion (10.3%). In the case of the SiO_2_ support, the catalyst deactivated over time due to metal sintering, which resulted from the weak NiO–SiO_2_ interaction. On the other hand, in the case of the 8 wt.% Ni/Al_2_O_3_ catalyst, the high surface area of the support (161 m^2^/g) led to low Ni dispersion (3.1%) due to SMSI-induced formation of a NiAl_2_O_4_ spinel phase. This phase transformation led to a higher carbon formation rate in the order of 16.3 mgC g_cat_^−1^ h^−1^. Modification of an alumina support with MgO led to higher Ni dispersion (7.6%) and a higher support surface area (S.A. 142 m^2^/g); the higher dispersion compared to the alumina counterpart was attributed to MgO, which was responsible for weakening the NiO–alumina SMSI caused by the reaction between Mg^2+^ and unsaturated Al^3+^ ions. This facilitated the reduction of the NiO species on the surface, and enhanced the activity and stability. As a result, 8 wt.% Ni/MA exhibited the lowest carbon formation rate (1.7 mgC g_cat_^−1^ h^−1^). On the other hand, 8 wt.% Ni/TiO_2_ exhibited the highest carbon formation rate of 24.7 mgC g_cat_^−1^ h^−1^ amongst the six catalysts. This was attributed to the low surface area (7 m^2^/g) and low Ni dispersion (2.0%). The MgO- and ZrO_2_-based catalysts presented low dispersion of 3.0% and 4.4%, respectively, due to their low surface areas of 23 and 21 m^2^/g, respectively. However, the former catalyst (4.2 mgC g_cat_^−1^ h^−1^) had a substantially lower carbon formation rate than the latter catalyst (21.0 mgC g_cat_^−1^ h^−1^). This quick deactivation of the 8 wt.% Ni/ZrO_2_ catalyst was attributed to the weak metal–support interaction (WMSI), which also caused the high initial activity observed. On the other hand, the anti-coking capabilities of the 8 wt.% Ni/MgO catalyst were attributed to the basic sites on the surface of MgO, which attract the acidic CO_2_ molecules. This study concluded that 8 wt.% Ni/MA had the best performance and stability amongst the six catalysts. 

Another study performed by Naeem et al. [193] studied Ni-based catalysts supported on three different supports—γ-Al_2_O_3_ (180 m^2^/g), CeO_2_ (40 m^2^/g), and ZrO_2_ (35 m^2^/g)—for DRM. All of the catalysts were synthesized via polyol methods. The authors concluded that the Ni-based catalyst supported on zirconia performed the best amongst the three catalysts because of its high reducibility (redox potential), surface acidity, and thermal stability. Meanwhile, Ni supported on a ceria catalyst had the highest carbon formation, which was attributed to the low Ni dispersion (large particle size). For the 5wt.%Ni/γ-Al_2_O_3_ catalyst, the coke inhibition was attributed to the basic surface of alumina and the high Ni dispersion and small Ni particle size.

### 5.2. Zeolites

Zeolites are a compelling group of aluminosilicate materials to consider for DRM reactions [120,121], as they have well-defined pores, high surface area, and an affinity for CO_2_. Zeolites can be classified into different groups based on their pore size—namely, microporous zeolites, which have pore sizes below 2 nm (<2nm); mesoporous zeolites, which have a pore size ranging between 2 and 5 nm [194]; and hierarchical zeolites, which can combine different levels of porosity. Mesoporous zeolites are among the most popular for the DRM reaction, as discussed below. The other important property of zeolites—apart from their porosity—that tunes their catalytic activity is their acidity; the latter can be tuned through the Si:Al ratio, either through the synthesis protocol or through post-synthesis processes. Generally, zeolites with a higher Si:Al ratio perform better in DRM because they are more basic. The better performance exhibited by zeolites with higher Si:Al ratios is correlated with higher coke and sintering resistance, due to the confinement of active metal inside the pores of the zeolites [118]. It was found that promoting zeolite supports with noble metals affects the stability of the active metal positively [153]. However, it has an infinitesimal effect on the conversion [161].

Jesus et al. investigated the catalytic performance of a Ni-based catalyst supported on beta zeolite for DRM [195]. Different loadings of MgO (0, 5, 10, and 20 wt.%) were deposited on the NH_4_-beta zeolites with a SiO_2_:Al_2_O_3_ molar ratio of 24.5 via the incipient wetness impregnation method. Ten-percent Ni was impregnated on the MgO-beta zeolite supports. The presence of Mg improved the catalytic performance of the catalysts and improved the conversion rates of CO_2_ and CH_4_, along with the stability and the H_2_:CO ratio. The catalyst with 10% MgO showed the best performance and the lowest carbon deposition (2.0 mg/gcat h) after 7 h of DRM at 800 °C. Najfach et al. synthesized Ni (10 wt.%) monometallic and bimetallic 10 wt.% Ni–X wt.% Mn (X = 5, 10, and 15) catalysts supported on commercial zeolites—namely, NH_4_-ZSM (Si/Al = 50, 425 m^2^/g), NH_4_-Y (Si/Al = 12, 730 m^2^/g), and Na-Y (Si/Al = 5.2, 900 m^2^/g)—and investigated the effect of Mn on the Ni-based catalysts’ DRM performance [196]. The addition of Mn improved the carbon resistance of the catalysts. The optimal Mn loading was related to the surface properties of zeolite. In particular, in the case of the 10 wt.% Ni/NH_4_-Y catalyst, the addition of 10 wt.% Mn improved the dispersion of Ni and enhanced the catalyst’s carbon resistance and activity in 24 h DRM. On the other hand, the addition of different Mn loadings (5 wt.%, 10 wt.%, and 15 wt.%) to the 10 wt.% Ni/ZSM catalyst had no significant effect on the performance. Furthermore, the addition of Mn to the Ni catalysts on the ZSM supports improved the stability of the catalysts, and a slight increase in Ni size was noticed. However, Mn decreased the initial conversion. The use of Na-Y zeolite compromised the stability of the catalyst during DRM. Loading of 10 wt.% and 15 wt.% Mn was found to facilitate the destruction of the Na-Y zeolite under a reducing environment. Luengnaruemitchai et al. compared the performance of X wt.% Ni-based (X = 3, 5, and 7) catalysts supported on various types of zeolites—zeolite A, zeolite X, zeolite Y, and ZSM-5—prepared by incipient wetness impregnation for DRM [197]. The Ni/zeolite Y exhibited the best performance amongst the catalysts. The following CH_4_ conversion trend was observed: Ni/zeolite Y > Ni/zeolite X > Ni/ZSM5 > Ni/zeolite A [197]. Moreover, the catalysts with 7 wt.% Ni loading showed the best performance compared to the other Ni loadings examined (3 and 5 wt.%), but higher carbon formation. Fakeeha et al. examined the stability of zeolite-supported Ni catalysts during DRM [88]. Amongst the three catalysts (5% Ni/γ-Al_2_O_3_, 5% Ni/Y-zeolite, and 5% Ni//H-ZSM-5) synthesized via the incipient wetness impregnation method, 5 wt.% Ni/H-ZSM-5 catalysts showed the best catalytic performance, stability, and carbon inhibition. Alotaibi et al. studied the effects of La and Ca as promoters for Ni-based catalysts supported on two different zeolite supports (NH_4_-Y) with different SiO_2_:Al_2_O_3_ ratios (5.1 and 12), denoted as Ni/ZY (A) and Ni/ZY(B), respectively [198]. The zeolite with the higher Si:Al ratio exhibited better catalytic performance than its investigated counterpart, but lower stability. Ni/ZL (B) and Ca- promoted Ni/ZL (B) catalysts showed the highest methane conversion, whereas the Ni/ZY(A) and Ni/ZY(B) catalysts promoted with La showed more time-on-stream stability, but lower catalytic performance. Ca and La promoters enhanced the stability of Ni-based catalysts.

### 5.3. Mesoporous Silicas

**Figure 17:**
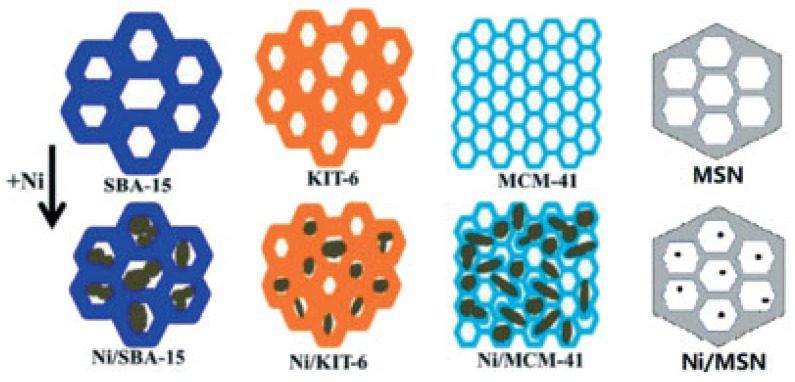
Confinement of active metal sites in mesoporous materials. Adapted from Ref. [224].

Various studies have been performed on mesoporous supports for DRM [199,200,201,202,203,204,205]. Mesoporous-silica-based zeolites have an inert surface with amorphous skeletal structures, with mesoscopic structures and adjustable pore sizes [206]. The weak surface acidity of mesoporous silicas—such as KCC-1, MCM-41, MSN, SBA-15, and KIT-6 (Figure 17)—reduces the carbon formation on the surface during DRM when these silicas are used as supports for Ni catalysts. 

Among the most popular silicas, SBA-15 has a thermally stable mesoporous structure with two-dimensional (2D) hexagonal channels [207,208]. Zhang et al. [209] were able to enhance the stability of a Ni/SBA-15 catalyst during DRM by confining the Ni particles inside the pores of the catalyst, preventing agglomeration. After 600 h, the catalyst was deactivated due to coke formation, but not metal sintering. Additionally, El Hassan et al. [210] compared Co supported on SiO_2_ and SBA-15 during DRM. It was found that the Co monometallic catalyst supported on SBA-15 had good dispersion of Co active sites; in particular, HR-TEM images showed good dispersion in the case of Rh_0.5_Co_12_/SBA-15, and occlusion of Co_3_O_4_ nanoparticles in the case of Co_12_/SBA-15. In addition, Kaydouh et al. performed a study on 5% Ni/SBA-15, and examined the effects of varying the Ni precursor (nitrate, chloride, and acetate) on the dispersion of Ni active sites and their location (i.e., inside or outside the pores of the SBA-15 support) [211]. The study deduced that there are no major effects on the textural properties; however, noticeable changes were observed in the size and location of the Ni active sites when varying the metal precursor. The catalysts with better metal confinement had better catalytic performance and stability for DRM. Rodriguez-Gomez et al. discovered that the formation of a nickel silicate phase in the inner surface of Ni/SBA-15 using in situ XPS and XAS techniques results in well-dispersed Ni particles with SMSI after reduction [212]. On the other hand, Ni/SBA-15 catalysts synthesized via traditional impregnation method exhibited low sintering and coking due to the confinement of Ni active sites [213,214]. 

MCM-41 is equally popular as SBA-15, exhibiting tunable porosity. Tian et al. synthesized a Ni-based catalyst using MCM-41 as a support [215]. The preparation method used by Tian et al. was able to generate monodispersed Ni nanoparticles with an average size of 2 nm and evenly distributed in the channels of the mesoporous support (SMSI phenomenon). The confinement of the Ni active sites reduced sintering and reduced carbon formation at high temperatures during DRM. However, the Ni nanoparticles on the surface of MCM-41 deactivated within 12 h of DRM. Additionally, Ni-MCM-14 synthesized via a microwave-assisted one-pot method resulted in smaller Ni nanoparticles than the ones synthesized via the wet impregnation method (Ni/MCM-41) [216]. In both catalysts, the mesoporous MCM-14 was synthesized from rice husk ash (RHA) via hydrothermal methods. The conversion rates of CH_4_ and CO_2_ were similar in both catalysts. However, the Ni-MCM-41 catalyst showed higher stability than its counterpart. A H_2_:CO ratio of 0.8 was obtained over the Ni-MCM-41 during DRM, proving the occurrence of an RWGS reaction [216]. Meanwhile, a H_2_/CO ratio of 1.1 was obtained during DRM over the Ni/MCM-41 catalyst, with higher formation of H_2_ via a methane decomposition reaction. Recently, silicon-rich wastes such as RHA have motivated researchers to synthesize silicon nanoparticles via a greener route [217,218]. The Ni-MCM-41 catalyst synthesized from RHA had no acidity [219], eliminating the need to add a basic modifier. Wang et al. introduced Mg to a Ni-based catalyst, which improved the surface basicity of the Ni/Mg-MCM-14 catalyst, along with the chemisorption and activation of CO_2_ [220]. Another study performed by Taherian et al. synthesized a Ni catalyst supported on MgO-modified MCM-41 via a one-pot method, and promoted it with Y_2_O_3_ to enhance the Ni activity [221]. The authors observed similar conclusions to Wang et al. [220], where the addition of Mg improved SMSI, whereas the addition of Y_2_O_3_ affected the catalyst’s reducibility positively. Ibrahim et al. [222] investigated the effects of different metal promoters—namely, x = Cs, Gd, Sc, Ce, and Ga—on the performance of a 5% Ni-x%/MCM-41 catalyst during DRM. The study showed that the catalysts promoted with Gd, Ga, and Ce exhibited the highest activity and carbon formation resistance, while Cs and Sc had adverse effects on CH_4_ and CO_2_ conversions. Xu et al. used alcohol during the impregnation of Ni on an MCM-41 support [223]. Five catalysts were prepared using five different alcohols, namely, methanol, ethanol, n-propanol, n-butanol, and ethylene glycol. The use of alcohols facilitated the movement of Ni^+2^ species into mesoporous channels of the MCM-41 support. This phenomenon was confirmed using FTIR, which showed a redshift in the 3445 cm^−1^ band in the alcohol-promoted catalysts’ spectra. This resulted in Ni size reduction. The smallest Ni size was observed with the catalyst synthesized by ethylene glycol, and consequently resulted in the best catalytic performance and carbon resistance during DRM. 

### 5.4. Layered Double Hydroxides (LDHs)

**Figure 18:**
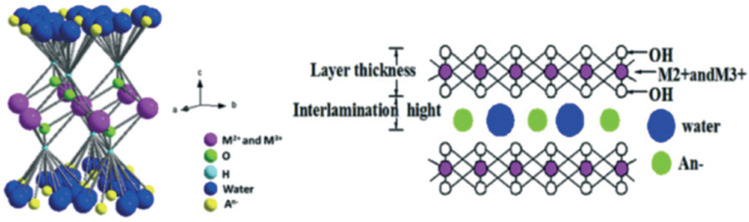
Schematic of the LDH structure. Reprinted with permission from Ref. [244]. Copyright 2015 Elsevier.

Layered double hydroxides (LDHs) with brucite-like layers are a series of two-dimensional (2D) materials [225,226,227,228,229]. Usually, LDHs consist of a positively charged layer (metal ions) and an anion layer, with water between the layers, as depicted in Figure 18, with a general formula of [M_1−x_^2+^-M_x_^3+^(OH)_2_]^x+^(A^n−^)_x/n_mH_2_O. Their unique structure, stability, facile synthesis, and low cost have prompted interest in the catalysis field [230,231,232]. LDHs have numerous superior properties that can enhance catalytic performance during DRM while preventing coking, namely, (i) a confinement effect, which generates small, monodispersed metal active sites; (ii) an SMSI effect, which prevents sintering and carbon formation; (iii) a memory effect in their structure, which makes the structure restoration (regeneration) possible using an anionic solution [233]; (iv) facile transformation of hydrotalcite to mixed oxides upon calcination [233]; and the fact that (v) synergistic new active sites can be engineered when LDH is synthesized with other materials [234]. (vi) Additionally, their large interface domain prevents metal agglomeration and maintains high metal dispersion [235]; and (vii) the surface basicity of the LDH layer can be tuned by changing the composition and ratio of cations, which affects the amount of hydroxyl groups [236]. Overall, LDH-derived catalysts are ideal for DRM, as they have high surface area, uniform active metal dispersion, and tunable active sites. The aforementioned characteristics of LDHs lead to highly active LDH-derived catalysts for DRM. The intrinsic features of the LDH-based catalysts can be easily tuned by inserting active metals between their interlayers. The dispersion of the active metal can be controlled by tuning its layer charge density [226]. Transition metal ions that have similar ionic radii to Mg^+2^ and Al^+3^ can be easily incorporated into the LDH structure, as described by Jiang et al. [237]. 

Dębek et al. synthesized a series of hydrotalcite-derived Ni–Mg–Al mixed oxides using co-precipitation, and varied the Ni content via substitution of 5, 15, 25, 50, 75, and 100% of Mg^2+^ cations in the brucite-like layers. The physiochemical properties of the prepared catalysts were investigated, along with their catalytic performance for DRM [238]. The study showed that reduction of the synthesized catalysts with 3%H_2_-Ar at 900 °C caused phase segregation of the Ni phase and the formation of Ni/NiO_x_ particles. The Ni–Mg–Al mixed oxides with 25% Ni content exhibited the highest basicity and the highest amount of strong basic sites, showing the highest CO_2_ conversion. Catalysts with higher Ni content showed higher carbon formation, which was attributed to the larger particle size of Ni, which is directly correlated with the Ni content. In another study, Zhu et al. synthesized hydrotalcite-derived Ni–Mg–Al mixed oxides with different Mg:Al ratios. The hydrotalcite-derived catalyst with a Mg:Al ratio equivalent to 1 exhibited the highest catalytic activity during DRM [239]. The study performed by Huang et al. showed that the catalytic activity toward DRM was directly associated with the LHD precursor used to synthesize the Ni–Mg–Al metal oxide [240]. The catalyst prepared using a freeze-dried LDH precursor was significantly more active and stable than that prepared with a bake-dried LDH precursor. Zhao et al. took advantage of the memory effect of LDHs to intercalate MoO_4_^−2^ anions into Fe/Mg/Al LDH flakes [241]. This resulted in a thermally stable catalyst (900 °C) and the growth of a double-helix CNT. Meanwhile, Świrk et al. compared the performance of Ce- and Y-promoted LDHs with pristine hydroxide (HT) catalysts for DRM [242]. The presence of Ce and Y promoters decreased the size of the Ni metal active sites, which consequently improved the catalytic performance during DRM. Bu et al. synthesized a stable catalyst using hexagonal boron nitride (h-BN) confined between the interfaces of LDH-derived Ni catalysts [243]. The catalyst’s stability was attributed to the SMSI and the confinement effect of h-BN/(Ni, Mg)Al_2_O_4_ sheets.

### 5.5. Metal–Organic Frameworks (MOFs)

Metal–organic frameworks (MOFs) are also known as metallated porous coordination polymers (PCPs), and they have numerous advantageous properties that enable their exceptional performance in heterogeneous catalysis [245], such as tunable framework, hybrid composition, defined and diverse crystal structures, etc. [246,247,248]. The use of MOF-based catalysts is comparatively new to DRM, but with a promising future. 

The high temperature window of DRM operation imposes limitations in the use of MOFs due to their low thermal stability. Recently, MOFs have been used as precursors to synthesize DRM catalysts. Karam et al. used an MOF with a high surface area (S_BET_ = 1130 m^2^ g^−1^) as a sacrificial template [249]. They used an Al-containing MIL-53 MOF and calcined the material to prepare a nickel–alumina-based catalyst (Ni^0^/Al_MIL_) with a spinel phase with Ni NPs embedded into an Al_2_O_3_-based matrix. Then, the obtained material was reduced, which resulted in the uniform dispersion of small Ni^0^ NPs on the lamellar γ-Al_2_O_3_. The confinement of Ni active sites in interlamellar spaces and the very high surface area led to additional stabilization of Ni active sites, which therefore inhibited CNT formation during DRM. The catalytic performance of the Ni^0^/Al_MIL_ catalyst was compared with that of the Ni^0^/γ-A_l2_O_3_ (Ni^0^/Al) catalyst. Ni^0^/Al_MIL_ displayed higher conversion rates of CH_4_ and CO_2_ than Ni^0^/Al. Additionally, Ni^0^/Al_MIL_ produced syngas with a H_2_:CO ratio close to 1, which is close to the thermodynamic threshold; this value means that fewer side reactions occurred during DRM. On the other hand, Ni^0^/Al resulted in a H_2_:CO ratio below 1, which was attributed to the simultaneous occurrence of an RWGS reaction. Furthermore, Ni^0^/Al_MIL_ showed outstanding stability after 100 h of reaction [249]. 

**Figure 19:**
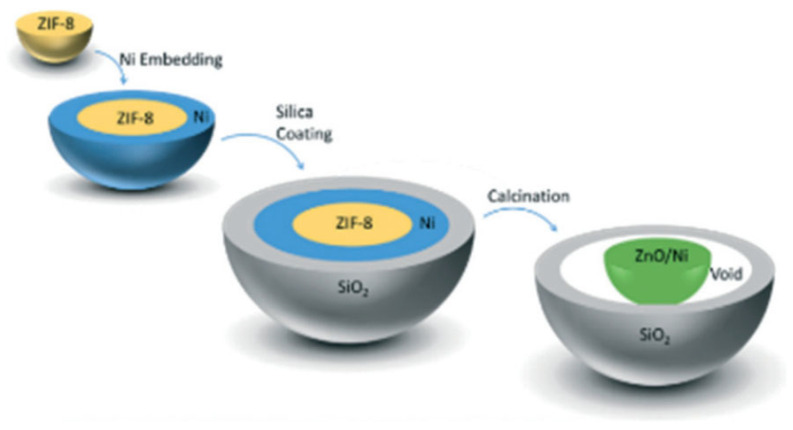
Schematic illustration of the preparation procedure followed to obtain the ZnO/Ni@m-SiO_2_ core–shell catalyst. Adapted from Ref. [250].

Similarly, in another study by Price et al., ZIF-8 was used as a sacrificial template to synthesize mesoporous ZnO/Ni@m-SiO_2_ yolk–shell particles [250]. Initially, Ni was impregnated on ZIF-8 MOF, forming a core-shell material. Then, a mesoporous silica shell was coated on the ZIF-8@Ni. Finally, the obtained material was calcined to yield a ZnO/Ni active metal core and a mesoporous SiO_2_ shell, and the ZIF-8 MOF was removed. The preparation procedure followed by Price et al. is schematically illustrated in Figure 19. The core–shell structure encapsulates the metal active sites, which prevent sintering of Ni active sites during DRM, while the shell blocks coke formation (i.e., acts as a barrier). 

Chin et al. also used MOFs for DRM [251], and varied the molar ratio of Ni:Ce (1:1, 2:1, and 1:2), with a total ratio of 4.5 mmol. The MOF-derived catalysts were synthesized via solvothermal methods. Ce promotion improved the Ni dispersion and resulted in small Ni particle size. Furthermore, the catalytic studies showed that the Ce-rich catalyst (Ni:Ce = 1:2) exhibited the highest conversion rates and stability.

In contrast to the previously mentioned studies, Vakili et al. used MOFs as supports rather than as sacrificial templates in plasma-assisted DRM [252]. This was accomplishable because the reactants were activated by electron activation rather than thermal activation. This did not compromise the framework of the UiO-67 MOF used with high surface area (2000 m^2^/g), and its intrinsic properties were maintained when treated in plasma conditions (discharge power, treatment time, and gas). The used UiO-67 MOF along with the dispersed Pt NPs (PtNP@UiO-67 catalyst) facilitated the plasma formation, and improved the CH_4_ and CO_2_ conversion rates. 

## 6. Density-Functional Theory (DFT)

Density-functional theory (DFT) is a powerful tool to provide deeper insight into the surface events and heterogeneous reaction mechanisms [253]. However, DFT does not eliminate the need for experimental testing, and it is computationally expensive—especially for reactions with a large number of intermediate species, such as Fischer–Tropsch reactions. DRM reaction mechanisms over Ni-based catalysts have already been extensively studied experimentally. Numerous studies stress the importance of approximating the network energies of catalytic surfaces to bridge the gaps in the DFT data [254,255,256,257,258,259]. Bligaard et al. created an open-source website (Catalysis-Hub.org) with DFT calculations on surfaces [260,261]. Currently, the website has 110,816 surface reactions available; however, not all of the reaction energies and barriers are reported. The website uses publications to grow or update its incomplete database. Other machine learning methods can be used to give better adsorption energy models, since DFT gives an approximate solution [259,262,263]. Bayesian inference can be used to derive reaction models from experiments [264]. 

Watwe et al. investigated the stability and reactivity of CH_x_ species on the Ni(111) facet [265]. The DFT calculations performed along with the kinetic analysis concluded that CH_x_ species prefer threefold sites. Additionally, CO and CH are the most abundant species on the surface. The adsorption of CH_3_ and CH_2_ species on Ni(111) was studied by Michaelides and Hu [266,267,268]. The DFT studies conducted showed that the sites of chemisorption for CH_3_ and CH_2_ depend on the three-center bonding of C-H-Ni. The DFT calculations performed by Zhu et al. to investigate the mechanisms of DRM summarized the most favorable metal–surface interaction [269]. The CO_2_ decomposition and H-induced CO_2_ decomposition were calculated to be 2.13 eV and 2.40 eV, respectively. According to the calculations, CO_2_ dissociated most dominantly into CO and O, and then the atomic O oxidized the CH_x_ intermediates. 

**Figure 20:**
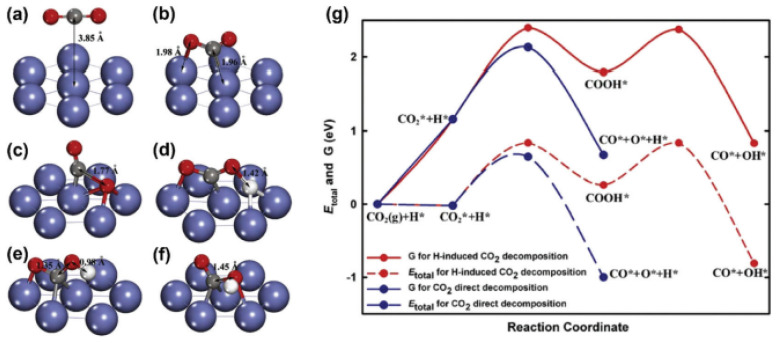
Geometries of (**a**) the physisorbed CO_2_ on Ni(111), (**b**) the chemisorbed CO_2_ on Ni(111), (**c**) the transition state for CO_2_ decomposition via the direct pathway, (**d**) the transition state for the H-inducted CO_2_ decomposition, (**e**) the intermediate for the H-induced CO_2_ decomposition, and (**f**) the transition state for COOH dissociation to generate CO and OH, along with the (**g**) total energy and Gibbs free energy diagrams for the CO_2_ direct decomposition and H-induced CO_2_ decomposition on Ni(111). Reprinted with permission from Ref. [270]. Copyright 2009 Elsevier.

As mentioned previously, DRM is a highly endothermic reaction (2.56 eV). This is because the reactants (CO_2_ and CH_4_) in this reaction are quite stable molecules. Hence, more energy is required to activate these stable molecules. Zhu et al. studied CO_2_ activation via direct decomposition (CO_2_ → CO + O) and H-induced CO_2_ decomposition (CO_2_ + H → COOH → CO + OH) on a Ni(111) surface. The DFT calculations performed showed that the direct decomposition route is more energetically favorable than H-induced decomposition [269]. All of the CO_2_ geometries of the initial states, intermediates, and transition states for CO_2_ decomposition (both direct and H-induced) are shown in Figure 20a–f. Figure 20g depicts the total Gibbs free energy and free energy diagrams for the two CO_2_ activation routes studied. Some studies also investigated direct CO_2_ decomposition on Ni(111) and Ni(211) surfaces [270,271,272,273], while other DFT studies were conducted on other metal surfaces to study CO_2_ activation [274,275]. Ko et al. performed a detailed DFT study on a series of monometallic and bimetallic alloy surfaces. A linear correlation was observed for the reaction heat ΔE(CO_2_^δ−^) of CO_2_ dissociation with respect to the sum of the adsorption heats of CO and O when the scaling relationship was applied on pure monometallic surfaces [274]. On the other hand, the Brønsted–Evans–Polanyi (BEP) relationship showed a linear relationship for the reaction heat ΔE(CO_2_^δ−^) of CO_2_ dissociation with respect to CO_2_^δ−^ dissociation, showing a combination of mixing rule, scaling relation, and BEP relationship used to quickly estimate the activation energies of CO_2_ dissociation over monometallic and bimetallic surfaces. The energy barrier increased from left to right and top to bottom of the grid. The Co-, Ru- Fe-, Ir-, Rh-, and Ni-based bimetallic alloys and Ir, Ru, Co, Ni, Rh, and Fe monometallic surfaces exhibited low activation energies (c.a. 0.75 eV), whereas Co-, Ru-, Ni-, Rh-, Ir-, and Cu-based bimetallic alloys and Pd, Pt, and Cu monometallic surfaces exhibited moderate activation energies (0.76–1.50 eV). The highest (1.51 eV) activation energies were observed for Au and Ag monometallic and Pd-, Pt-, and Cu-based bimetallic alloys.

## 7. From Funded Research to Technological Innovation

The DRM reaction has been the subject of many patents in an effort to develop the best catalyst possible that can bridge the lab results with the industrial need for practicality. Among the handpicked patents in the open literature, some present promising results—particularly from a materials design perspective. In particular, Takanabe et al. [276] reported on a boron-treated cobalt catalyst that seemed to be superior compared to the traditional compositions. The systems investigated—Co/ZrO_2_, Co/CeO_2_, and Co/CeO_2_-ZrO_2_—were treated with NaBH_4_, keeping NaBH_4_/Co at 6.3. The catalysts showed conversions > 65% (CH_4_) and 75% (CO_2_) for 20 h on stream, with <0.01 wt.% deposited carbon. The non-boron catalyst had conversion values < 30%. Due to the added value of having boron as a promoter in the structure, different B-sources were synthetically tested—namely, NaBH_4_ and NH_3_BH_3_. In all of the catalysts prepared with varying B:Co ratios, the carbon deposition amount was small. The patent by Ko et al. [277] stresses the use of perovskite-type oxides; in the structure, Ni, Fe, or Co is partially substituted for the Ti site (B-site) in the case of SrTiO_3_, MgTiO_3_, CaTiO_3_, or BaTiO_3_. The catalytic material was tested for different methane reforming reactions (e.g., steam, dry) as well as partial oxidation. In the experiment, traditional catalysts were used, such as 10 wt.% Ni/alumina, as well as 10 wt.% Ni/0.5Ru/Mg/alumina. The perovskite-based catalysts presented low or no carbon deposition; thus, these catalysts can be good substrates for industrial use. Another promising catalyst formulation was proposed with a core–shell structure [278]. Intermetallic compounds have also been proposed as viable catalysts for the DRM reaction [279]. The latter materials can contain Ni/Sn, among other compositions, and they can be used as electrodes or catalysts, minimizing the size requirements for a potential device while suppressing the coking. Other patents, from a process perspective, report on two-dimensional catalysts and, in particular, DRM, along with carbon recovery [270] or continuous dry reforming processes for high-quality syngas production in a fluidized bed reactor [279]. A method of coupling the DRM reaction with regeneration by using a composite catalyst was presented by Wu et al. [280]. Using a composite catalyst containing CaCO_3_ (NiO-CaCO_3_/Al_2_O_3_), the problem of the calcium carbonate decomposition being limited by high temperature was addressed, while the CO_2_ produced by the calcium carbonate decomposition was utilized in the DRM process. A plethora of catalyst compositions and architectures have also been patented as very promising candidates for the DRM reaction, such as carbides [281], SiO_2_@NiCe core–shell structures [282], Ni–mesoporous silica anti-coking and anti-sintering catalysts [283], hollow zeolites with bimetallic and trimetallic active metals [284], metal ferrites [285], etc., highlighting the importance of material design to the process efficiency and viability. 

## 8. Concluding Remarks and Future Perspectives

DRM is one of the CO_2_ utilization methods, and it can be considered as one of the contributors to a circular carbon economy, as it is a tool for returning CO_2_ to the fuel production line. Overall, there is an imperative need to improve the materials design and understand the mechanistic pathways of the reaction and the regeneration/recovery cycles. Attaining insights into DRM will allow us to mature the materials design so as to tackle issues such as sintering and coking, both of which are currently bottlenecks for the industrialization of the process. From the materials design perspective, the active metal, support, promoter, structure, and methods of preparation and activation are crucial factors. Due to the perplexity of their effects on the ultimate catalyst activity, there is always an observed tradeoff between merits and drawbacks. As discussed in this review, SMSI is an important contributor to the DRM reaction. SMSI can lead to the formation of solid solutions either between metal and the support (e.g., RhMg_2_O_4_) or between the metal and the promoter, (e.g., NiO-MgO). This can have controversial outcomes, spanning from metal protection against sintering (activity increase) to loss of exposed active metal (activity decrease). In addition, the metal particle size and metal dispersion can affect the catalytic activity and stability. Improving the dispersion (i.e., small metal catalyst) can be achieved through intensifying the SMSI, or by enhancing the metal oxide’s reducibility (e.g., through nanostructuring or doping). 

The dispersion and particle size of the metal, along with the basicity, oxygen storage capacity, reducibility, porosity, and surface area of the support, are all crucial factors for DRM. The acidity/basicity of the supports is another pivotal property for DRM, since the CH_4_ decomposition takes place on the metal and acid sites of the supports, while the activation of the mildly acidic CO_2_ takes place on the basic supports. Activation of CO_2_ can also be controlled through the oxygen storage capacity of the supports. The mobile oxygen contributes to the cleaning of the surface from the deposited carbon as the mobile oxygen species actively participate in carbon’s oxidation to CO/CO_2_.

On the other hand, the reducibility of the support can tune the number of active sites. Strong metal–support interaction can induce the metal’s decoration with a support entity (MO_x_), leading to encapsulation (reduction) of active sites. These phenomena are usually driven by the reduction (pre-treatment) conditions applied. On catalysts with an intrinsically poor reducibility, noble metals or more reducible supports should be added. Catalysts with well-defined architectures usually exhibit high specific surface area, and a uniform and interconnected pore network.

According to this review, a great potential for the design of DRM catalysts can be envisioned; current/new MOFs can be seen to bear important traits, such as tunable metal sites and chemical flexibility in terms of functional groups. At the same time, the metal oxide catalysts remain at the heart of the DRM catalysis, but there is a need for a more controlled synthesis so as to be able to manipulate critical features such as metal particle size, porosity, acidity/basicity, metal–support interface, and coking tendency. Since the metal–support interface is of great importance, affecting the electronic and geometric interaction of the phases involved, the use of MOFs as sacrificial templates can provide tunability to the metal oxide fabrication.

From the mechanistic perspective, it is important to understand the CH_4_ and CO_2_ activation and the energetics of the process involving both experimental and computational tools. Parameters such as binding energies, bonding angles, bonding lengths, charge transfer, and the adsorption site (i.e., close to the dopant site or further) are key to understand the CH_4_/CO_2_–surface interaction. Particular emphasis needs to be given to the use of advanced characterization tools that will allow us to probe the mobility of the lattice oxygen (e.g., transient isotopic exchange studies), and to evaluate the surface events in real time (e.g., SSITKA). In addition, X-ray techniques (e.g., EXAFS, XANES) will allow us to discuss true bimetallicity issues, and how the change in the metal’s electronic state impacts on the surface catalysis.

## Data Availability

Not applicable.
